# Bone-conduction hyperacusis induced by superior canal dehiscence in human: the underlying mechanism

**DOI:** 10.1038/s41598-020-73565-4

**Published:** 2020-10-06

**Authors:** Xiying Guan, Y. Song Cheng, Deepa J. Galaiya, John J. Rosowski, Daniel J. Lee, Hideko Heidi Nakajima

**Affiliations:** 1 Department of Otolaryngology-Head and Neck Surgery, Harvard Medical School, Boston, MA USA; 2 Massachusetts Eye and Ear, Boston, MA USA; 3 New York University Medical Center, New York, NY USA; 4 Otolaryngology-Head and Neck Surgery, Johns Hopkins University School of Medicine, Baltimore, MD USA

**Keywords:** Diseases, Medical research

## Abstract

Our ability to hear through bone conduction (BC) has long been recognized, but the underlying mechanism is poorly understood. Why certain perturbations affect BC hearing is also unclear. An example is BC hyperacusis (hypersensitive BC hearing)—an unnerving symptom experienced by patients with superior canal dehiscence (SCD). We measured BC-evoked sound pressures in scala vestibuli (*P*_*SV*_) and scala tympani (*P*_*ST*_) at the basal cochlea in cadaveric human ears, and estimated hearing by the cochlear input drive (*P*_*DIFF*_ = *P*_*SV*_ – *P*_*ST*_) before and after creating an SCD. Consistent with clinical audiograms, SCD increased BC-driven *P*_*DIFF*_ below 1 kHz. However, SCD affected the individual scalae pressures in unexpected ways: SCD increased *P*_*SV*_ below 1 kHz, but had little effect on *P*_*ST*_. These new findings are inconsistent with the inner-ear compression mechanism that some have used to explain BC hyperacusis. We developed a computational BC model based on the inner-ear fluid-inertia mechanism, and the simulated effects of SCD were similar to the experimental findings. This experimental-modeling study suggests that (1) inner-ear fluid inertia is an important mechanism for BC hearing, and (2) SCD facilitates the flow of sound volume velocity through the cochlear partition at low frequencies, resulting in BC hyperacusis.

## Introduction

Bone conduction (BC) hearing is commonly used for practical purposes: standard hearing tests measure BC thresholds to assess sensorineural hearing; BC hearing aids help patients with conductive and mixed hearing loss; and BC earphones of various types are increasingly popular. However, the underlying mechanism of BC sound transmission to the inner ear has been elusive and poorly understood because BC sound transmission is complex—multiple frequency-dependent mechanisms may be involved^1–3^. An understanding of BC has been further hindered due to the inability to measure BC sound transmission directly in the inner ear. However, recent developments now allow the experimental measurement of intracochlear sound pressures during BC in fresh human cadaveric specimens^4,5^, which can provide detailed information regarding inner-ear sound transmission and estimate hearing function. Perturbations of the normal system, such as by superior canal dehiscence (SCD), can further aid in understanding BC sound transmission mechanisms.

Clinically, SCD is a pathological opening of the bony capsule surrounding the superior semicircular canal that can cause various vestibular and auditory symptoms. SCD symptoms include sound- or pressure-induced dizziness, autophony, pulsatile tinnitus, reduced hearing for air conducted sound, and increased sensitivity to bone conducted sound, known as BC hyperacusis^6,7^. BC hyperacusis manifests as a pathological awareness of self-generated sounds (one’s own heartbeats, voices, footsteps, joint movements, chewing, eye movements)^7–10^ as well as particular external sounds and vibrations (doors slamming, machinery, noise experienced inside an automobile)^11–13^. SCD patients can display a supra-normal BC threshold on audiometric testing^6,7,9^.

While the association between SCD and its characteristic influence on BC are widely recognized, how SCD results in BC hyperacusis is not well understood. Although the effect of SCD increasing BC hearing has been simulated using various computational models^1,14,15^, the BC mechanisms upon which those simulations were built are inconsistent. Importantly, none of these models have been validated against detailed quantitative experimental measurements that can provide information about BC sound transmission within the human inner ear.

In the present study, we measure intracochlear sound pressures in scala vestibuli (*P*_*SV*_) and scala tympani (*P*_*ST*_) at the cochlear base of fresh human cadaveric temporal bone specimens. This allows us to determine the basal cochlea’s differential pressure (*P*_*DIFF*_ = *P*_*SV*_ − *P*_*ST*_), which has been shown to have the same frequency–response characteristics as an evoked sensory potential, the cochlear microphonic, measured in living animals^16,17^. *P*_*DIFF*_ represents the cochlear input drive that starts the traveling wave, and can be used to estimate hearing^18^. Evidence has shown that ear pathologies such as ossicular disarticulation and SCD produce changes in audiometric air-conduction (AC) thresholds measured in patients that are consistent with changes in *P*_*DIFF*_ measured in temporal bones^18–20^. Here we measure AC- and BC-elicited *P*_*SV*_, *P*_*ST*_, and *P*_*DIFF*_ before and after creating an SCD in the specimen. AC stimulation is generated by a speaker placed in the ear canal; BC stimulation is generated by a bone-anchored hearing aid (Baha) mounted on the temporal bone. To understand the mechanism by which SCD affects the response to BC, we create a lumped-element inner-ear model based on a theory of inner-ear fluid inertia. All the impedances that make up the parameters in the model are experimentally derived directly from our present AC measurements and previous measurements conducted during round window (RW) stimulation^21,22^. The intracochlear pressure measurements verified with the modeling analysis provide a new understanding of BC sound transmission and the mechanism underlying SCD-induced BC hyperacusis.

## Results

### During AC, SCD decreases *P*_*SV*_, *P*_*ST*_ and *P*_*DIFF*_

We previously determined the effects of SCD on AC-evoked intracochlear pressures^19,23–25^. AC *P*_*SV*_, *P*_*ST*_ and *P*_*DIFF*_ measurements in the present specimens confirmed that the SCD effects were as previously reported^19,23–25^—SCD decreased AC-evoked *P*_*SV*_, *P*_*ST*_ and *P*_*DIFF*_ below 1 kHz (Supplementary Fig. S1). Previously, we demonstrated an understanding of the SCD effects on AC responses by matching experimental measurements with computational modeling^19,25^. Similar AC modeling simulations are described for the present experiments in the Supplementary Information (Figs. S2 and S4).

To develop our new BC computational model, we used our new AC measurements to characterize the impedances of inner ear structures in the present group of ears in the manner previously described^21^. The acoustic impedances of the cochlear partition (*Z*_*DIFF*_), RW (*Z*_*RW*_), and superior semicircular canal with SCD (*Z*_*SC*_) of each individual ear were determined from the present AC measurements (see Supplementary Figs. S1 and S3); their median values are listed in the Supplementary Table S1 and are used in the BC model described below. Representative parameters of the reverse middle-ear impedance at the oval window (*Z*_*ME*_) and the vestibular aqueduct impedance (*Z*_*VA*_)—previously derived from our earlier RW-stimulation experiments^21,22^—are included in Supplementary Table S1 and are used in our BC model.

### During BC, SCD increases *P*_*SV*_ and *P*_*DIFF*_

**Figure 1:**
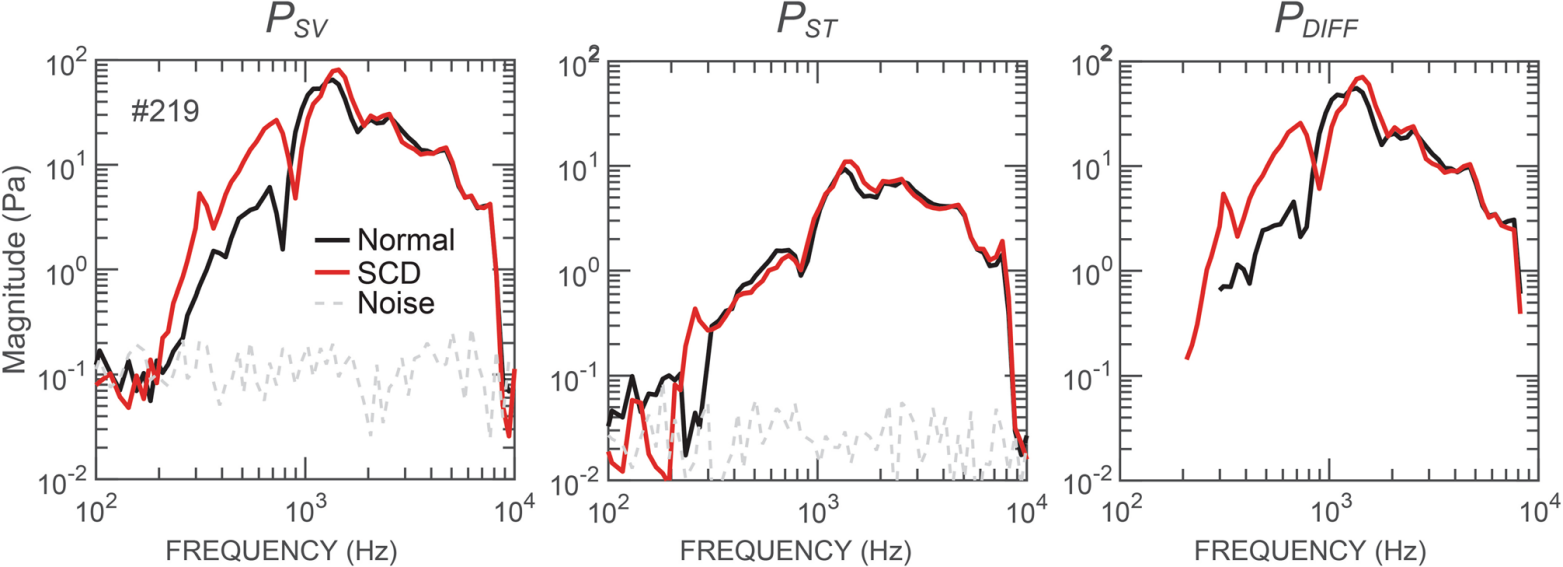
Magnitude frequency responses of *P*_*SV*_, *P*_*ST*_, and *P*_*DIFF*_ during BC stimulation in a representative ear. Black lines are the pressures under the normal condition, red lines are the pressures after an SCD was produced, and grey dashed lines are the noise floor of the pressure sensor. *P*_*SV*_ and *P*_*ST*_ with a signal-to-noise ratio < 6 dB were omitted in the calculation of *P*_*DIFF*_.

Figure 1 plots the BC-evoked *P*_*SV*_*, P*_*ST*_*,* and *P*_*DIFF*_ magnitudes under normal (black) and SCD (red) conditions measured from a representative ear (#212). Results from all the individual ears are plotted in the Supplementary Fig. S5.

We found that during BC, SCD generally (five of the six ears studied) increased *P*_*SV*_ below 1 kHz. Interestingly, the effects on *P*_*ST*_ were generally smaller. The resulting effect of SCD on *P*_*DIFF*_ was an increase below 1 kHz. One ear, #211, exhibited little change in the measured pressures (Supplementary Fig. S5).

**Figure 2:**
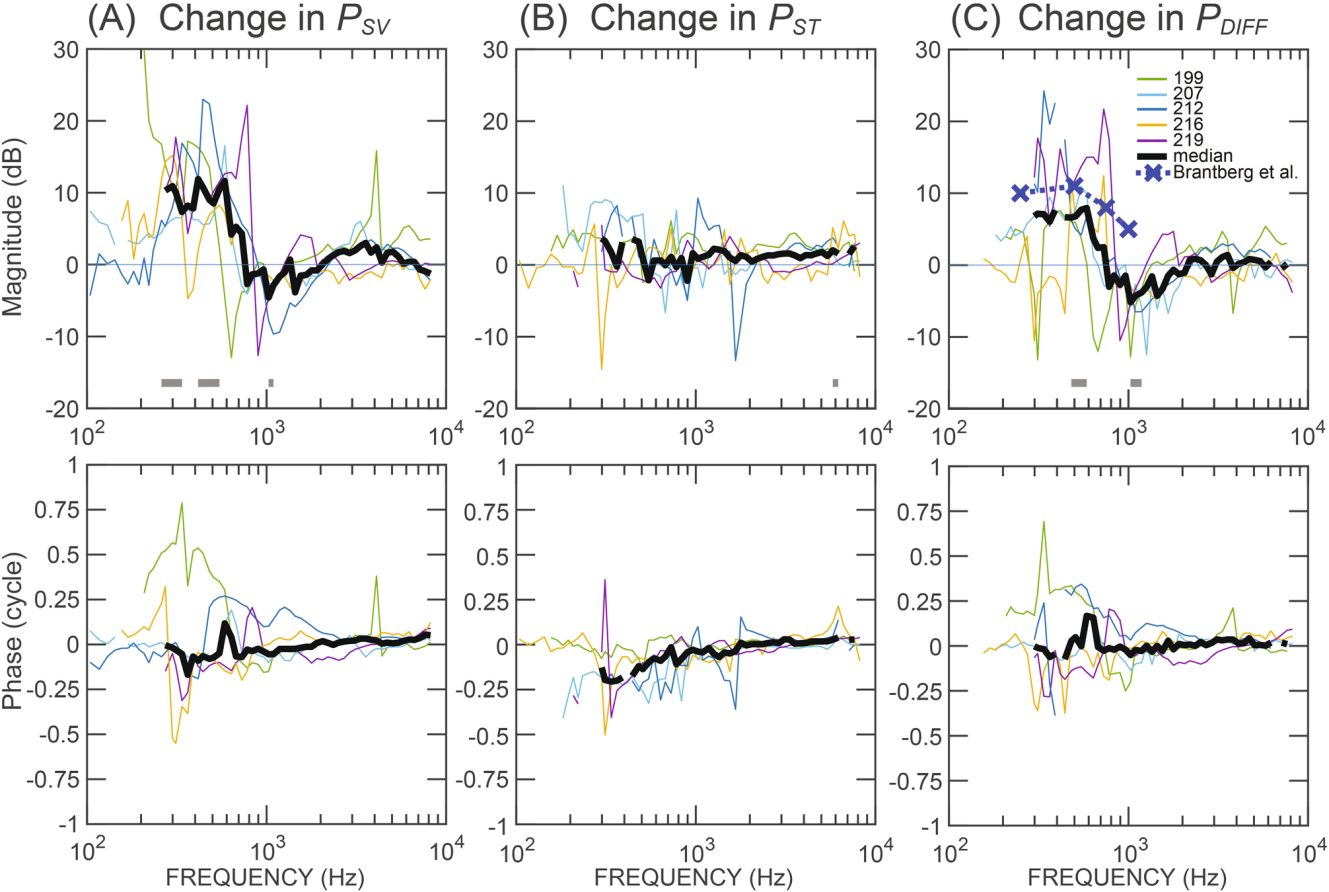
SCD-induced changes in magnitude and phase of (**A**) *P*_*SV*_, (**B**) *P*_*ST*_, and (**C**) *P*_*DIFF*_. Colored lines represent the change in five individual ears; for each individual ear only the intracochlear pressure data with signal-to-noise ratios greater than 6 dB are included. Black lines represent the median of those five ears; the median is not shown at frequencies where the sample size is less than five. The gray-colored horizontal bars near the x-axis represent the frequency range where the changes are significant (p < 0.05). Blue dashed line with crosses in (**C**) represents the increased sensitivity to BC stimulation reported in SCD patients^9^.

Figure 2 plots the SCD induced changes on the magnitude and phase of the BC-elicited *P*_*SV*_, *P*_*ST*_, and *P*_*DIFF*_ for the five ears. The colored lines represent the pressure changes due to SCD in individual ears; the black lines represent the median. The magnitude of the change in *P*_*DIFF*_ is determined by $20log_{10} (|P_{{DIFF\_SCD }} /P_{{DIFF\_Normal }} |)$, where *P*_*DIFF_Normal*_ is the differential pressure *P*_*SV*_ − *P*_*ST*_ under the normal condition and *P*_*DIFF_SCD*_ the SCD condition.

As shown in the upper panel of Fig. 2A, SCD in general increased *P*_*SV*_ magnitude by 5–20 dB below 1 kHz, but with varied effect size and frequency range in the five ears. Statistically significant changes were found between 250 and 550 Hz, as indicated by the horizontal gray bars near the x-axis. In a narrow bandwidth around 1 kHz, SCD also significantly decreased *P*_*SV*_ by as much as 10 dB. The median of this reduction was smaller compared to individual data due to variation in the frequency where this decrease occurred across ears. At higher frequencies, *P*_*SV*_ did not change much. In contrast, SCD only produced a small increase in *P*_*ST*_ (median < 5 dB) below 500 Hz, but this change was not statistically significant (upper panel of Fig. 2B).

The estimate of hearing—*P*_*DIFF*_—increased by 5–20 dB below 1 kHz, decreased by ~ 10 dB in narrow bandwidths in the range of 0.6–1.5 kHz, and did not change much at higher frequencies (upper panel of Fig. 2C). These changes were statistically significant at 500–600 Hz and near 1 kHz. To compare with clinical audiometric data, average BC threshold of 10 SCD ears relative to that of 10 normal ears reported by Brantberg et al.^9^ is plotted as the dark blue thick dashed line with the cross symbols in Fig. 2C (Brantberg et al. only reported SCD-induced threshold changes at frequencies of 1 kHz and lower.). The SCD-induced increase in BC hearing at low frequencies in patients is similar to the increase in *P*_*DIFF*_ we measured in our temporal bone preparation.

As shown in the lower panels of Fig. 2, SCD in general did not affect the phase of *P*_*SV*_, *P*_*ST*_, and *P*_*DIFF*_ in BC, except in one ear (#199) where SCD increased the phase of *P*_*SV*_ and *P*_*DIFF*_ by 0.25–0.5 cycles below 600 Hz.

### BC models based on inner-ear bone compression are inconsistent with experimental measurements as they predict that SCD decreases *P*_*SV*_

Several past reports suggest the mechanism behind SCD-induced low-frequency BC hyperacusis is inner-ear bone compression^15,26^. To test this mechanism against our new experimental data, we built a model that simulates bone compression (Supplementary Fig. S6). With this model, it was possible to simulate SCD-induced increase in *P*_*DIFF*_ at low frequencies (Supplementary Fig. S7). However, the bone-compression model could only produce such changes in *P*_*DIFF*_ by greatly reducing *P*_*SV*_ compared to *P*_*ST*_, a change that is contrary to our experimental observations.

### A BC model based on inner-ear fluid inertia theory successfully simulates the effect of SCD on BC inner-ear sound transmission

During BC, the bony inner ear wall undergoes rigid-body motion in which the entire bony wall vibrates with the same amplitude and phase^1,14^. This motion of the bone induces entrained motions to the enclosed inner-ear fluids and is thought to be the main drive in BC inner-ear fluid inertia^27–29^. In this theory, the entrained motion produces sound pressures inside the inner ear by accelerating the inner-ear fluid. Although it has been a point of speculation for a long time, the fluid-inertia mechanism lacked a clear mathematical or physical definition. Recently, Stenfelt^1^ proposed a method to calculate the sound pressure produced by a volume of fluid that is under bulk sinusoidal movement. He estimated the inner-ear sound-pressure sources generated by the bulk motion of the inner-ear fluid^1^. Here, we describe our BC inner-ear fluid-inertia model built upon Stenfelt’s calculation of inertia-induced pressure sources, and demonstrate that the model predicts the effects of SCD on *P*_*SV*_, *P*_*ST*_, and *P*_*DIFF*_ that are generally consistent with our experimental findings.

**Figure 3:**
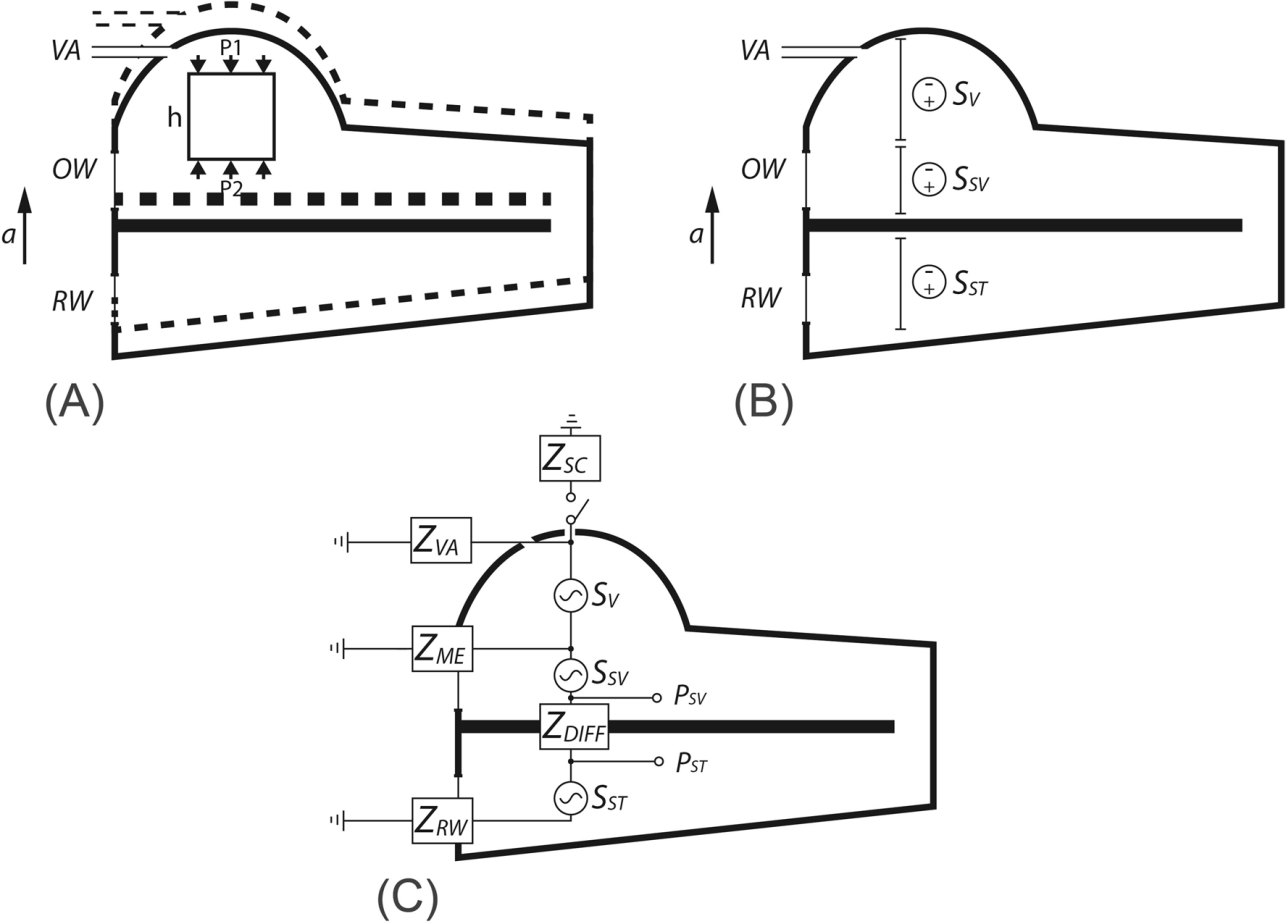
(**A**) Illustration of BC rigid-body motion of the inner-ear wall. The dashed-line boundary represents the position of the inner-ear wall at an instant away from the temporal origin. The thick line within the bony wall represents the basilar membrane. An arrow indicates the direction of acceleration. The square box represents a free-body diagram of the inner-ear fluid for use in analyzing the relation between the pressure and acceleration (Eqs. 1–3). (**B**) The three vertical sections designated by double-ended lines represent sound-pressure differences due to equivalent sound sources produced by the bulk motion of the fluid across the vestibule (*S*_*V*_), scala vestibuli (*S*_*SV*_), and scala tympani (*S*_*ST*_). (**C**) The lumped-element circuit model for BC sound transmission driven by the fluid-inertia sound sources.

Figure 3A illustrates the concept of rigid-body motion of the inner-ear wall in BC. For simplification, we focus on the direction of the motion perpendicular to the basilar membrane (represented by the thick horizontal line in Fig. 3A). For the upward portion of the cycle of the rigid-body motion, the inner-ear wall translates from one location (the solid-line boundary) to another (dashed-line boundary) without deformation. The fluid in the inner-ear thereby undergoes a bulk motion with amplitude and phase synchronized to the bony wall. To demonstrate how this motion produces sound pressure inside the inner ear, a free-body diagram of a small volume of fluid is drawn and shown as the box in Fig. 3A. The height of the box is *h*, and the area of its top or bottom surface is *A*. When the entire inner-ear fluid is accelerating upwards, in light of Newton’s second law, the net force on the fluid in the free-body diagram in the vertical direction is

1 $$F = \rho hAa,$$

where $\rho$ is the density of the fluid and *a* is the acceleration of the fluid. Assuming the viscosity of the fluid is negligible, the net force is related to the pressures on the top and bottom surfaces by

2 $$F = (P2 - P1)A$$

Subsequently,

3 $$P2 - P1 = \rho ha$$

As described by Stenfelt^1^, Eq. (3) indicates that there is a drop of pressure in the fluid across a given height in the direction of the motion. If we divide the space of inner-ear fluid near the cochlear base into three sections—the vestibule (V), scala vestibuli (SV), and scala tympani (ST)—there is a pressure drop across each section shown as *S*_*V*_, *S*_*SV*_, and *S*_*ST*_, respectively, in Fig. 3B. When the inner-ear fluid is accelerating upward, for each of those three sections, the pressure at the bottom is greater than that at the top (Eq. 3). Figure 3C shows how simple harmonic vibration of the inner-ear fluid would make the corresponding pressure drops sinusoidal, and how those pressure drops would interact with our lumped circuit parameter description of the inner ear. The key hypothesis for this BC inner-ear fluid-inertia theory is that those sinusoidal pressure drops—*S*_*V*_, *S*_*SV*_, and *S*_*ST*_—serve as sound *pressure sources* and generate net volume velocities between the mobile windows and leakage channels in the inner ear^1,2^.

We tested how SCD affects *P*_*SV*_, *P*_*ST*_, and *P*_*DIFF*_ in the lumped-element model shown in Fig. 3C. The three sources in the model—*S*_*V*_, *S*_*SV*_, and *S*_*ST*_—were determined by the simple physical principles of Eq. (3), where the heights *h* of the simplified fluid compartments—the vestibule, SV, and ST—are 5.8 mm, 1.2 mm, and 1.4 mm, respectively (as in^1^). The density of the fluid $\rho$ is 1000 kg/m^3^, and the acceleration *a* is 1 m/s^2^. As previously mentioned, all the impedances in the model were experimentally derived and are detailed in Supplementary Table S1.

**Figure 4:**
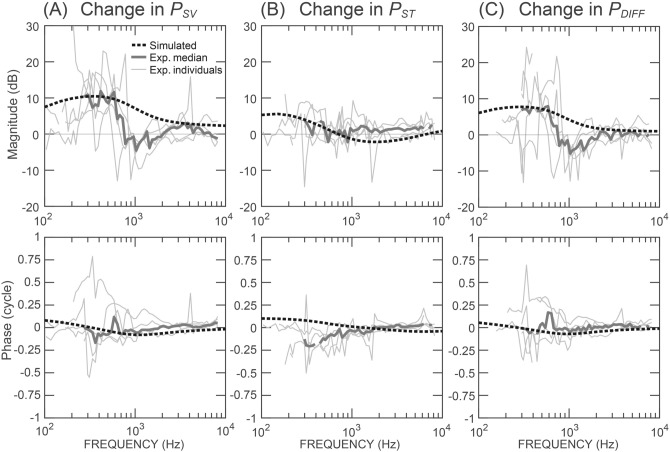
Changes in magnitude and phase of *P*_*SV*_, *P*_*ST*_, and *P*_*DIFF*_ due to SCD, simulated by the fluid-inertia model (dashed lines) compared with the temporal-bone measurements. The thin gray lines represent measurements of individual ears; the thick gray lines represent the median of the individual results.

Figure 4 plots the SCD-induced changes in the magnitude and phase of BC-elicited *P*_*SV*_, *P*_*ST*_, and *P*_*DIFF*_ simulated from our fluid-inertia model (thick dashed lines), and directly compares these model results to our measurements in temporal bone experiments. As shown in the upper panels of Fig. 4, the model predicts that SCD increases the magnitudes of *P*_*SV*_ by 8–12 dB and *P*_*DIFF*_ by 7–9 dB below 1 kHz, with smaller increases in *P*_*ST*_ below 500 Hz. Similar magnitude changes can be seen in the pressure measurements from temporal bone experiments. As shown in the lower panels of Fig. 4, the model predicts that the phases of *P*_*SV*_, *P*_*ST*_, and *P*_*DIFF*_ are not affected much by SCD, which also agrees with the temporal bone measurements.

A minor inconsistency is that the model-predicted increases in *P*_*SV*_ and *P*_*DIFF*_ take place over a slightly wider frequency band (up to about 2 kHz) compared to the experimental measurements (up to 1 kHz). A possible explanation is that above 1 kHz, other sources of BC-induced sound pressures are active in the experiments, but not included in our model, such as inertia of the middle-ear ossicles^30^. These additional sources may generate additional *P*_*SV*_ and *P*_*ST*_. Another possible explanation for the inconsistency is that our BC inertia model only simulates motion of the inner ear in one dimension (motion perpendicular to the basilar membrane in the base), but the 1 kHz minimum in the measurements may represent the contribution of the effects of motion in other directions.

Our BC inertia model provides an explanation for the SCD’s varying effects on *P*_*SV*_ and *P*_*ST*_ at low frequencies observed in Fig. 4A,B. In the model, *P*_*SV*_ and *P*_*ST*_ depend on the *S*_*ST*_ source, the impedances *Z*_*DIFF*_ and *Z*_*RW*_*,* and *U*_*DIFF*_, the volume velocity (acoustic current) that flows across the cochlear partition:

4 $$P_{SV} = S_{ST} + (U_{DIFF} Z_{DIFF} ) + (U_{DIFF} Z_{RW} )$$

5 $$P_{ST} = S_{ST} + (U_{DIFF} Z_{RW} )$$

On the SV side of the cochlea, because *Z*_*DIFF*_ is quite high, *U*_*DIFF*_ * Z_*DIFF*_ is the dominating influence on *P*_*SV*_. As indicated by the increase in *P*_*DIFF*_ (*P*_*DIFF*_ = *U*_*DIFF*_ * Z_*DIFF*_), SCD increases *U*_*DIFF*_, which significantly elevates *P*_*SV*_. In contrast, on the ST side, *Z*_*RW*_ is so small that the constant inertial source *S*_*ST*_ dominates *P*_*ST*_. Therefore, the increase in *U*_*DIFF*_ due to SCD does not much affect *P*_*ST*_, consistent with our temporal bone experimental measurements.

## Discussion

The temporal bone experiments in the present study show that for BC, SCD increases *P*_*DIFF*_ at low frequencies, consistent with clinically measured changes in audiometric thresholds seen with patients that experience BC hyperacusis^7,9^. Our measurements point out that the increase in *P*_*DIFF*_ (increase in hearing) results from the independent effect on the individual scalae pressures—*P*_*SV*_ is increased whereas *P*_*ST*_ is little affected. The differing effects on the two scalae pressures contradict some of the theoretical models that have been used to explain SCD-induced BC hyperacusis.

Previously, three BC models have been used to explain BC hyperacusis induced by SCD—one based on the inner-ear bone compression mechanism and two based on the fluid inertia mechanism^1,14,15^. Although these models each predict an increase in *P*_*DIFF*_, the studies did not report the individual effects in *P*_*SV*_ and *P*_*ST*_ due to SCD. We built models with circuit arrangements similar to those in Rosowski et al.^15^ and Stenfelt^1^, but found that the simulated changes in *P*_*SV*_ and *P*_*ST*_ predicted by these previous models were inconsistent to our experimental findings. For example, in the Supplementary Information, we detail how SCD in the model built upon the bone compression mechanism can result in increase in *P*_*DIFF*_. However, the increase in *P*_*DIFF*_ results from the changes in *P*_*SV*_ and *P*_*ST*_ that differ from our experimental measurements (Supplementary Fig. S7). The third study, by Kim et al.^14^, uses a finite-element model. We do not have access to this model and therefore were unable to test its predictions to compare to our experimental results.

Simulation of our new BC fluid inertia model presented here generally captures the SCD effects on *P*_*SV*_, *P*_*ST*_, and *P*_*DIFF*_ observed in temporal bone experiments. This consistency between model and experimental results is achieved by the combination of the model’s architecture, inertial pressure sources derived from theoretical studies, and impedance parameters that are completely determined experimentally from our temporal-bone measurements. The theoretical estimation of the fluid inertia sources are calculated as in Stenfelt^1^. The parameters for our BC model are not varied to “fit” the effect of SCD on BC. Rather, they represent the medians of the impedances determined from the data of AC experiments performed in the same ears (for *Z*_*DIFF*_, *Z*_*RW*_, and *Z*_*SC*_) and the averaged impedances from our previous RW-stimulation experiments by Stieger et al.^22^ and Frear et al.^21^ (for *Z*_*VA*_ and *Z*_*ME*_).

There are notable inter-individual variations in each of the measured impedances (Supplement Figs. S1 and S3 and Table S1). To test how sensitive the BC inertial model was to the variability of each impedance, we investigated how the simulated changes in *P*_*SV*_, *P*_*ST*_, and *P*_*DIFF*_ due to SCD were influenced as each of the model impedances was varied from its minimum to maximum (parameters defined in Supplemental Table S1) while the other impedances were held at their median or average values. The results are provided in Supplemental Figs. S8–S12.

In general, the significant low-frequency increase in *P*_*SV*_ and *P*_*DIFF*_ due to SCD was maintained as we varied the model impedances over their measured range, demonstrating the model is robust to impedance variations across ears. We also found that the simulated increases in *P*_*SV*_ and *P*_*DIFF*_ are more sensitive to the variations in *Z*_*SC*_, *Z*_*VA*_, and *Z*_*DIFF*_—as those impedances change from their minimum to maximum, one at a time, the low-frequency increase in *P*_*SV*_ and *P*_*DIFF*_ can vary by 5–10 dB (Supplemental Figs. S8–S10). In contrast, variations in *Z*_*ME*_ or *Z*_*RW*_ generally had limited effects on the simulation, except that using the maximum measured *Z*_*RW*_ in the model caused a notable change in the increase of *P*_*ST*_ (Supplemental Fig. S12B).

In patients with SCD, the impedance of the superior-canal branch, *Z*_*SC*_ (shown in Fig. 3C), represents the summed impedance of the anterior and posterior arms of the superior canal, the SCD, and the termination of the SCD made by the external content interfacing the surface of the SCD. Differences in SCD size, location, and the termination can affect the summed impedance, and potentially lead to different effects on hearing. Effects of size and location of SCD on AC hearing in human were investigated with temporal bone studies and modeling studies^14,15,19,23–25^. Temporal bone experiments showed that ears with SCD exhibited low-frequency AC hearing loss and that increases in SCD size caused more reduction in the AC hearing until the SCD size exceeded certain dimensions. However, it is also found that similar SCD sizes can produce varied reductions of hearing in different ears^23^, preventing prediction of SCD size by AC hearing loss. In addition, no difference in AC hearing was noted between anterior and posterior SCD locations of the superior canal^23^.

The effects of these SCD variables on BC hearing are unknown. We predicted how variations in the size and the termination of the SCD affected the intracochlear pressures in the BC inertia model by replacing *Z*_*SC*_ with a structure-based acoustic circuit (Supplemental Fig. S13). This allowed us to theoretically determine the total impedance of the circuit for different sizes of SCD and different termination conditions (Supplemental Table S2 and Figure S14).

The simulation suggests that when the diameter of a small circular SCD increases from 0.1 to 0.4 mm, the low-frequency increases in the BC-evoked *P*_*SV*_, *P*_*ST*_, and *P*_*DIFF*_ become more prominent (Supplemental Fig. S15). However, when the diameter of the circular SCD is 0.4 mm or greater, the changes in *P*_*SV*_, *P*_*ST*_, and *P*_*DIFF*_ are insensitive to further size increase of the dehiscence (Supplemental Fig. S15). We also found that the increases in *P*_*SV*_, *P*_*ST*_, and *P*_*DIFF*_ for a given SCD size remain the same when we changed the SCD termination from a fluid-filled cranial vault (i.e. brain) to an air-filled middle-ear cavity.

The model in Fig. 3C provides an explanation of how fluid inertia contributes to BC hearing. To further test this mechanism, other perturbations to the system could be studied in future temporal bone experiments. For example, we could immobilize the oval window and RW to possibly reduce the net volume velocity passing through the cochlear partition, hence to remove the contribution of *Z*_*DIFF*_ and *Z*_*RW*_ on *P*_*SV*_ and *P*_*ST*_ during BC stimulation (Eqs. 4 and 5). With that, we could determine if *P*_*SV*_ and *P*_*ST*_ are proportional to the acceleration as assumed in Eq. (3).

Another possible mechanism to explain BC is that sound can be transmitted from the contents of the skull—brain, dura, and cerebrospinal fluid (CSF)—to the inner ear via conducting channels such as aqueducts, nerves, and vasculature^31–33^. With SCD, the BC sound produced in the cranial tissues within the skull may be transmitted to the inner ear directly through the dehiscence. This pathway is thought to be a possible cause of certain SCD-induced symptoms of BC hyperacusis, such as hearing one’s eyeballs move^7^. Although the present study shows that BC can be increased by SCD in isolated temporal bones, BC-induced sound pressures within the cranium of the whole head^33^ may lead to additional sound transmission from the cranium to the inner ear via SCD and thus further enhance BC. To model this intra-cranial sound conducting mechanism, a sound pressure source representing the sound in the skull may be added atop the SCD in Fig. 3C. To test such mechanism and determine the intracranial pressure drive, bone-conduction studies in whole-heads could be used to simultaneously measure intracranial and intracochlear pressures.

In our isolated temporal bone experiments, a small amount of fluid was maintained atop the SCD to prevent air entering the inner ear. The Baha-induced vibration of the bone supporting this fluid might have generated sound, which could propagate through the fluid reservoir and through the dehiscence, entering the inner ear. This possible external source in the temporal bone experiment would work similarly as the sound sources in brain/CSF/dura in the whole head experiment. In the future, we can measure the sound pressure near the SCD to test for the presence of such mechanism.

Another limitation in our present study is the use of a commercial Baha for BC stimulation. Although the Baha is the most clinically relevant BC stimulator, it produces complex three-dimensional motion. Thus, the BC motion in our experiments would not be solely aligned in the manner depicted in Fig. 3A, where the motion is simplified as one dimensional to allow for simpler estimation of sound pressure sources. Theoretically, the magnitude of the pressure sources produced by fluid inertia would depend on the direction of the bulk movement of the fluid because the direction determines the effective height to which the pressure source is proportional. Previous finite-element modeling analysis suggests that each of the three orthogonal rigid-body inner-ear motion can produce *P*_*SV*_, *P*_*ST*_, and *P*_*DIFF*_^34^. Thus, the measured changes in intracochlear pressures due to SCD might be the summed effects of three orthogonal directions, which may explain the more complex frequency-dependent fluctuations (the sharp peaks and notches in the frequency responses) and the inter-ear variability observed in our experiments. Complex motion also makes it difficult to identify the relationship between the acceleration of the bone and intracochlear pressure measurements. It is possible that a shaker that generates one-dimensional vibration to the specimen might provide smoother frequency responses and clarify the fluid-inertia mechanism, although this type of one-dimensional vibration would not be realistic in a live human.

## Summary

We measured the effects of SCD on BC-elicited intracochlear sound pressures in SV and ST at the base of the cochlea in human cadaveric ears. SCD increased *P*_*SV*_ by 5–20 dB significantly between 250 and 550 Hz, but had limited effect on *P*_*ST*_. Such results were inconsistent with predictions of previous inner-ear BC models. We developed a new inner-ear model based on the inner-ear fluid-inertia concept proposed by Stenfelt^1^ and restricted our parameters to those derived from temporal bone experiments under AC and RW stimulation. Our new model was able to simulate the measured effects of SCD on *P*_*SV*_ and *P*_*ST*_ individually as well as the BC hearing estimated by the differential pressure across the cochlear partition, *P*_*DIFF*_. The model suggests that SCD produces hypersensitivity to BC sound, or BC hyperacusis, by increasing the flow of sound volume velocity through the cochlear partition.

## Methods

This study was approved by the Institutional Review Board of the Massachusetts Eye and Ear Infirmary. All the methods in this study were performed in accordance with the relevant guidelines and regulations. An informed consent was obtained from the donor or next of kin for the fresh human cadaveric specimens used in this research.

### Temporal bone preparation

23 human cadaveric temporal bones were used in the study. The specimens were prepared in the same manner as described in our previous studies^4,18,23,24^. Briefly, the temporal bones were removed within 24 h of death. After extraction, the specimen was either used for experiment within 1–2 days or immediately frozen for future use.

To prepare a specimen, the facial recess was widely opened to allow middle-ear vibration measurements and insertion of pressure sensors. The stapes tendon was sectioned and removed to allow access to the oval and round windows. The cochlear promontory near the oval and round windows was thinned to facilitate drilling of cochleostomies for insertion of pressure sensors into the SV and the ST. The transmastoid approach for the SCD was used. The epitympanic bone overlying the superior semicircular canal was thinned to facilitate the opening of an SCD.

To minimize large fluctuations in vibrational magnitude of the entire specimen across frequency during BC excitation, the temporal bone was placed in malleable putty (Play-Doh, Cincinnati, OH, USA) that cushioned the specimen to produce smoother frequency responses, yet remained mechanically stable for consistent AC- and BC-evoked vibration^4^.

### AC and BC stimulation

The setup for AC and BC stimulation was similar to that described in Stieger et al.^4^. For AC stimulation, a coupler for the speaker and calibrated probe-tube microphone was sealed to the bony rim of the ear canal. Pure-tone AC sounds, sweeping from 20 Hz to 10 kHz with 72 logarithmically distributed points, were presented to the ear canal with a speaker (model 40-1377, Radio Shack). The tip of the probe-tube microphone recording the stimulus sound was about 2 mm from the umbo of the tympanic membrane. The pure tone at each frequency was 50 ms in length and repeated 25 times for averaged response.

For BC stimulation, a Baha (BP100, Cochlear, Australia) was anchored to an abutment implant screwed to a thick area of bone at the mastoid near the ear canal. The exact location and relative direction of the anchoring screw varied slightly across experiments, but generally the screw was almost parallel to the bony ear canal. The processor of the Baha was programmed for a linear presentation, and driven with a sequence of sinusoidal voltage at 40 mV_RMS_ from 20 to 10 kHz with the same 72 frequency points as in AC stimulation. This level of voltage stimulation was confirmed for linearity across all frequencies used. At each frequency the input voltage to the Baha was 50 ms in length and averaged 50 times.

### Intracochlear pressure measurements

For intracochlear pressure measurements, ~ 220 µm diameter cochleostomies in the bone overlying SV and the ST at the basal cochlea were created by hand with a sharp pick while the entire cochlear promontory was submerged under saline to prevent air from entering the cochlea. While maintaining saline submersion, a 150–200 µm diameter fiber-optic sensor^18,35^ was inserted with micromanipulator through the cochleostomy until the tip of the sensor was 100–150 µm below the inner surface of the cochlear bony capsule. The tip of the sensor was close to the otic capsule and as far as possible from the cochlear partition so that the pressures measured would be dominated by the fast wave instead of the slow wave of the traveling wave^36^. After the insertion, the cochleostomy was hermetically sealed to the outer bony surface with alginate dental impression material (Jeltrate, Milford, Delaware, USA), which dries in wet environments to a rubbery consistency.

To reduce measurement artifact due to the relative motion between the sensor and bone during BC (as described above), the method described in Stieger et al.^4^ was used to ensure that the sensor was firmly glued to and vibrated with the bony surface. After the alginate which hermetically sealed the cochleostomies around the sensor was set, the saline around the sensor shaft was removed and the surrounding area completely dried. Then, dental cement (Durelon, 3M Corp.), which dries to a hard consistency, was applied over the alginate to tightly fix the sensor shaft to the bony surface. The sensor was then released from the micromanipulator, enabling the sensor to vibrate together with the otic capsule and cochlear fluid, and to prevent any leak at the cochleostomies.

### Vibration measurements of the stapes and round window

A small reflective tape was placed on the posterior crus of the stapes and on the center of the RW membrane. A single point 1D laser vibrometer (CLV 1000, Polytec) was used to measure the velocities of the stapes (*V*_*STAP*_) and round window membrane (*V*_*RW*_) during AC stimulation. The laser beam was focused on the reflective tapes in the measurements. The direction of the laser was close to the piston-motion directions of the stapes and the round window.

### Experimental protocol

Accuracy of intracochlear pressure measurement is important in calculating the differential pressure (computed from the complex difference between *P*_*SV*_ and *P*_*ST*_), especially because the magnitude of the sensitivity of a fiber-optic sensor can change during the experiment. To monitor the sensor sensitivity during the experiment, we developed a new intracochlear monitoring method described in detail in Stieger et al.^4^.

#### Baseline AC responses

Initial assessment of the specimen included using Laser-Doppler measurements to check for inner-ear air or leak by ensuring that AC-evoked velocity of the stapes (*V*_*STAP*_) and round window (*V*_*RW*_) had ½ cycle phase difference below 500 Hz, and that middle-ear velocities were normal^4^. We followed a protocol illustrated in the flow chart of Supplemental Fig. S16. The cochleostomies were made under saline. The fiberoptic pressure sensors were calibrated (C1) in a water vial attached to a shaker (4290, Bruel and Kjaer, Denmark). If sequential calibrations were stable within 0.2 dB across all frequencies, sensors were inserted into the cochlea and sealed with only alginate. AC-evoked *V*_*STAP*_ or *V*_*RW*_ were measured simultaneously with *P*_*SV*_ and *P*_*ST*_. Imperfect seals or introduction of air could easily occur, altering velocities of the stapes and RW and their phase relationship. The velocity responses were confirmed to be stable before and after insertion of sensor, which ensured that air did not enter the cochlea and there was no leak at the seal. Thereby, the first intracochlear pressure measurements (AC1) were made in the condition equivalent to a normal intact inner ear.

The fiberoptic sensors were then removed from the cochlea under fluid and recalibrated in the vial (C2) at least twice to confirm stable sequential recordings (difference < 0.2 dB). If the difference between this calibration (C2) and the initial calibration (C1) was less than 2 dB at all the frequencies, the sensor was considered to be stable and the intracochlear pressures measured at AC1 were used as references for future AC pressure responses.

After obtaining these AC reference pressures in the normal ear, the sensors were inserted into the cochlea again, sealed with a small amount of alginate, and cemented firmly to the promontory as described above. *V*_*STAP*_, *V*_*RW*_, and intracochlear pressures were measured again in response to AC stimulation (AC2). Repeatability of pressure measurements and stability of the velocities including the half-cycle relationship of stapes and RW velocities were checked to determine whether air entered the cochlea during the reinsertion of the sensors or if there was a leak. Usually, AC pressure responses measured at AC2 were within 2 dB of those at AC1. If a constant pressure magnitude shift and stable phase across all the frequencies occurred, a correction for the pressure sensor sensitivity could be performed with the AC reference pressure response AC1. If there was a frequency-specific change in pressure, then this shift was likely not a sensor sensitivity shift, but another issue (e.g. air entered the inner ear or a leak occurred).

#### BC responses

After AC2, *P*_*SV*_ and *P*_*ST*_ in response to BC stimulation were measured. To check if the vigorous vibration during BC changed the pressure sensor sensitivity and the integrity of the seals, AC responses were repeated after BC recordings (AC3) and compared with those recorded before BC recordings (AC2). If the AC responses before and after BC measurements were repeatable (within < 0.5 dB), the normal-ear AC and BC recordings were deemed reliable.

#### SCD

The method to create the SCD was similar to previous studies^23–25^. Using the transmastoid approach to the superior canal, the epitympanic cavity was filled with saline to cover the bone over the superior canal. The saline was prevented from entering the tympanic cavity by positioning the temporal bone and/or making a small dam with alginate between the epitympanic and tympanic cavities. A dehiscence with a length of approximately 1 mm was drilled at the center of the superior semicircular canal arc. Intracochlear pressures and velocities were then measured during AC, followed by BC and another AC. The repeated AC responses were to check for stability of the sensors.

We then reversed the effects of the SCD on the AC response by patching the dehiscence by overlying the dehiscence with a paper slightly larger than the size of the dehiscence followed by application of alginate. The intracochlear pressures and velocities of the stapes and RW during AC stimulation with the patched SCD were compared with those measured before the superior canal was opened (normal baseline). Reversal of the AC responses confirmed that (1) the changes in pressures or velocities observed after opening of the superior canal, if any, were indeed caused only by the SCD and (2) the pressure sensors’ sensitivities and seals were stable through the entire process, (3) air was not introduced into the inner ear, 4) other changes in the specimen did not occur.

The reason we used 1 mm dehiscence in this study is that our previous experience with SCD experiments showed that longer dehiscences are challenging to resurface well to completely reverse the effect of SCD. Furthermore, the increased SCD effect due to increase in size over 1 mm was small^23^.

### Summary of specimens used

This study used 23 fresh and freshly frozen temporal bones. 17 ears were excluded due to instability of the sensors, trauma during the experiment, or abnormal middle or inner ear such as low stapes velocity or the presence of air or leak in the cochlea.

In the 6 remaining specimens (from 3 males and 3 females, between the age of 23–87), the sensor sensitivities were well characterized throughout the experiment because the AC responses measured before and after the BC responses were repeatable for each measurement run, and the SCD effect on AC responses could be reversed by patching the dehiscence.

### Statistical analysis

The changes in BC-elicited *P*_*SV*_*, P*_*ST*_*, P*_*DIFF*_ in dB unit (Fig. 2) were tested for the null hypothesis using One-sample *t* test in Matlab. Statistical significance was defined as p < 0.05.

## Supplementary information

Supplementary Information.
