# Supplementary material for: Bone-conduction hyperacusis induced by superior canal dehiscence in human: the underlying mechanism
Source: Sci Rep. 2020 Oct 6;10:16564. doi: 10.1038/s41598-020-73565-4 (PMC7538896; doi:10.1038/s41598-020-73565-4)
Supplement: Supplementary file 1 — Supplementary Information. [file 41598_2020_73565_MOESM1_ESM.pdf]

Supplementary Information

Bone-conduction hyperacusis induced by superior canal dehiscence in human: the underlying mechanism

Xiying Guan[1,2*], Y. Song Cheng[1,2,3], Deepa J. Galaiya[1,2,4], John J. Rosowski[1,2], Daniel J. Lee[1,2], Hideko Heidi Nakajima[1,2]

[1]Department of Otolaryngology, Harvard Medical School, Boston, Massachusetts, USA
[2]Massachusetts Eye and Ear, Boston, Massachusetts, USA
[3]New York University Medical Center, New York, New York, USA
[4]Otolaryngology-Head and Neck Surgery, Johns Hopkins University School of Medicine, Baltimore, Maryland, USA
*email: xiying_guan@meei.harvard.edu

Air Conduction

Intracochlear pressures during AC stimulation in temporal bone experiments.

For the temporal bone experiments studying the effects of SCD on BC (results shown in Fig. 2 of main manuscript), AC pressure responses were also measured for normal condition and after SCD. The effects of SCD on intracochlear pressures during AC were consistent to earlier reports[1-3]: SCD resulted in P_{SV}, P_{ST} and P_{DIFF} decreasing at low frequencies (from thin solid to thin dashed lines in Fig. S1).

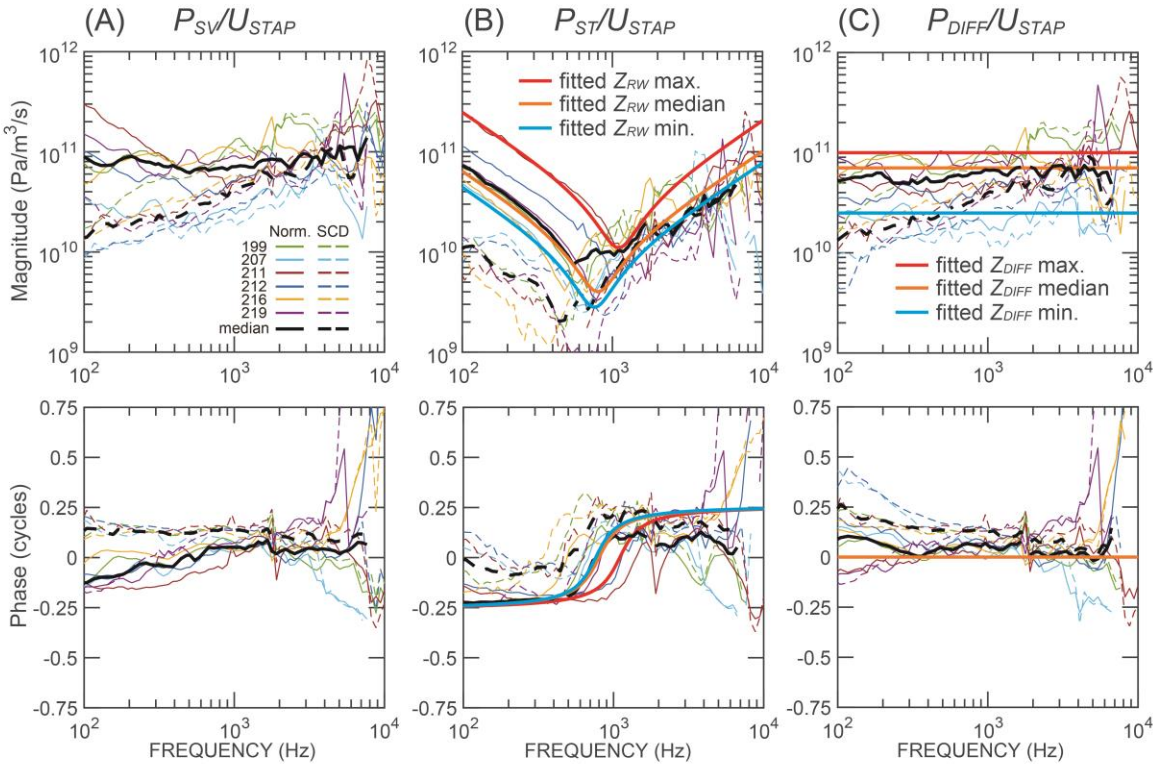

Figure S1. Magnitude and phase of (A) P_{SV} and (B) P_{ST} at the cochlear base normalized to the volume velocity of stapes footplate (U_{STAP}) during AC stimulation. The normalized differential

pressure or the cochlear drive (P_{DIFF}/U_{STAP}) is shown in (C). Pressures measured from individual ears are designated by color, with thin solid lines representing normal (before SCD) and thin dashed lines representing after SCD. The thick black lines represent the medians of the normal inner ear; the dashed black lines represent the medians after SCD. In (B) and (C), the thicker orange lines represent fitted impedances of RW (Z_{RW}) and cochlear partition (Z_{DIFF}) of the median curves of normal ears by using acoustic components and the parameters in the column 'Median Z' in Table S1. The thicker blue and red lines represent the fitted low and high boundaries, respectively, of the variations in Z_{RW} and Z_{DIFF}. The parameters used to fit the low and high boundaries of the impedance variations are listed in the columns 'Minimum Z' and 'Maximum Z' in Table S1. In this manner, the median impedance parameters for use in the computational model and the ranges of those parameters were experimentally determined.

Determining impedances of the inner ear from AC intracochlear pressure measurements. During AC stimulation, the effect of SCD on inner-ear sound transmission can be simulated by a lumped-element model with experimentally determined impedances from temporal bone experiments. Figure S2 shows the AC inner-ear model developed for AC sound transmission in the inner ear for normal and SCD conditions [1,4-7]. For a normal ear (switch open in Fig. S2), the volume velocity generated at the stapes footplate (U_{STAP}) flows through the cochlear partition (with impedance of Z_{DIFF} which includes the helicotrema) and exits the inner ear at the round window (with impedance of Z_{RW}). The sound pressures measured near the oval and round windows are P_{SV} and P_{ST}, respectively. For an ear with SCD, the superior canal forms a 'third-window' pathway (switch closed in Fig. S2). With SCD, part of U_{STAP} is shunted towards the superior semicircular canal (with impedance of Z_{SC}) and exits at the dehiscence, while the rest of U_{STAP} goes towards Z_{DIFF}.

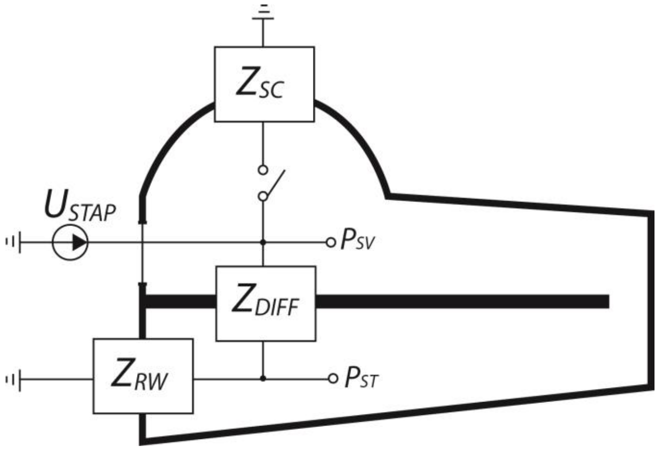

Figure S2. AC inner-ear model: Schematic of the lumped-element model for sound transmission in the inner ear during AC stimulation. Closing the switch represents an SCD.

In the normal inner ear of temporal bone experimental measurements:

$$Z_{RW} = P_{ST\_Normal} / U_{STAP\_Normal} \qquad \text{(Eq. S1)}$$
$$Z_{DIFF} = P_{DIFF\_Normal} / U_{STAP\_Normal} \qquad \text{(Eq. S2)}$$

Thus, the solid lines in Fig. S1B are Z_{RW} and the solid lines in Fig. S1C are Z_{DIFF} of our temporal bones.

In the SCD condition, impedance of the superior canal Z_{SC} is determined by

$$Z_{SC} = P_{SV\_SCD} / [U_{STAP\_SCD} - P_{SV\_SCD} / (Z_{DIFF} + Z_{RW})] \qquad \text{(Eq. S3)}$$

Figure S3 below plots Z_{SC} of individual ears - obtained from the measurements - with thin colored lines and their medians with thick black lines.

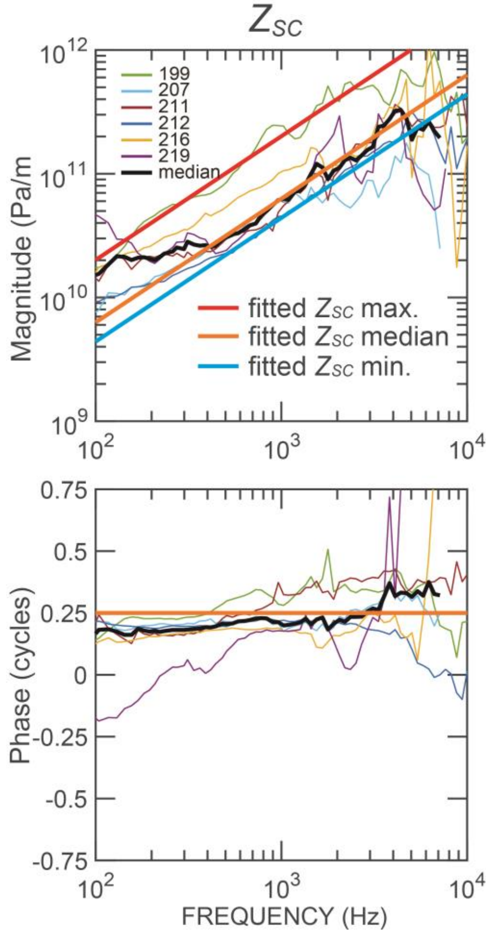

Figure S3. Magnitude and phase of the acoustic impedance of the superior semicircular canal with SCD (Z_{SC}). Thin colored lines represent the impedances obtained from the measurements of individual ears and the thick black lines represent the median. The thick orange line represents the fitted median of Z_{SC} using the parameter in the column "Median Z" in Table S1. The thick blue and red lines represent the fitted low and high boundaries of the variations in Z_{SC}, respectively. The parameters used to fit the low and high boundaries are listed in the columns 'Minimum Z' and 'Maximum Z' in Table S1, respectively.

To determine representative impedance parameters Z_{RW}, Z_{DIFF}, and Z_{SC}, we fitted the median of Z_{RW}, Z_{DIFF}, and Z_{SC} of the individual ears from the present study using acoustic elements

(acoustic compliance, mass, and resistance), as in our previous studies [1,4,6] and others[8,9]. The values of those components are provided in the column 'Median Z' in Table S1 below. In addition, representative parameters of the reverse middle-ear impedance at the oval window, Z_{ME}, and the impedance of the vestibular aqueduct, Z_{VA} – previously derived and published by our group[4,10] – are also provided in the column 'Median Z' in Table S1. These five experimentally derived impedances are used to test two types of BC models: the BC inner-ear compression model described below in this supplementary information and the BC inner-ear fluid-inertia model described in the main article.

The fitted median impedances are plotted as thick orange lines in Figs. S1B (Z_{RW}), S1C (Z_{DIFF}), and S3 (Z_{SC}). Note that the median of the measured Z_{RW} in Fig. S1B does not correctly reflect the sharp resonant compliance-to-mass transition seen in the individual ears because the frequency of this transition varies across ears. The fitted Z_{RW} has a relatively sharp transition, more representative of the individual measurements.

The inflection at 400 Hz seen in the median of Z_{SC} results from the Z_{SC} variations between the individual ears. Two out of six ears showed this inflection in Z_{SC} but the other four ears did not (Supplemental Fig. S3). Thus the low-frequency inflection is not a common feature of Z_{SC}. The most prominent pattern of Z_{SC} below 4000 Hz is an increase in magnitude with frequency at a rate of 20 dB/decade accompanied by a phase of + 0.25 cycle (i.e. positive 90 degree). This frequency dependence in our experimental results suggests that Z_{SC} resembles an acoustic mass, which is very likely contributed by the mass of the fluid in the superior canal. Thus, we used a single inertance component, L_{SC}, to fit the median of Z_{SC} $(= j\omega L_{SC})$.

The frequency dependence of Z_{SC} at f > 4000 Hz varies between ears. This variation may reflect complications in our estimates of stapes motion. The volume velocity of the stapes (U_{STAP_Normal}) used in the calculation of Z_{SC} (Eq. S3) is estimated from a single point 1D LDV measurement, but the motion of the stapes at the high frequencies in AC can be complex and three dimensional. Thus it is likely that there are errors in the measurement-derived Z_{SC} above 4000 Hz. Given the individual variations and the potential errors, we did not attempt to accurately fit Z_{SC} above 4000 Hz.

We also fitted the low and high boundaries of the inter-individual variations in those impedances, and the corresponding parameters are provided in columns 'Minimum Z' and 'Maximum Z', respectively, in Table S1. Those values were used to study the effects of parameter variation on the BC inertia model, and the relevant results are provided in the section 'Effects of parameter variation on the BC inertia model' below.

Table S1. Experimentally derived inner-ear element impedances for computational modeling. The parameters in 'Median Z' column were used in the AC and BC models to calculate the simulated results shown in Fig. 4 and Supplemental Figs. S4 and S7. Most values in the 'median' column fit within the ranges defined by Frear et al.[4] and Raufer et al.[6] with the exception of L_{RW} which is 10% larger than largest L_{RW} computed in the earlier study. Parameters were derived from the present experiments except for Z_{ME} and Z_{VA} that were obtained from previous experiments[4]. The parameters in columns 'Minimum Z' and 'Maximum Z' represent the limits of impedance variations across ears seen in Figs. S1 and S2 and the previous study[4] and were used to calculate the effects of parameter variation on the BC inertia model shown in Supplemental Figs. S8-S12.

Impedance	Composition	Element type and Units	Parameters		
			Minimum Z	Median Z	Maximum Z
Cochlear partition Z_{DIFF}	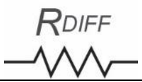	$R_{DIFF}\ (Nsm^{-5})$	2.50×10^{10}	$\mathbf{7.05 \times 10^{10}}$	10.00×10^{10}
Round window Z_{RW}	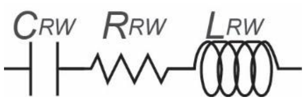	$C_{RW}\ (N^{-1}m^{5})$	3.54×10^{-14}	$\mathbf{2.45 \times 10^{-14}}$	6.37×10^{-14}
		$R_{RW}\ (Nsm^{-5})$	2.80×10^{9}	$\mathbf{4.00 \times 10^{9}}$	11.00×10^{9}
		$L_{RW}\ (Ns^{2}m^{-5})$	1.24×10^{6}	$\mathbf{1.61 \times 10^{6}}$	3.29×10^{6}
Superior canal Z_{SC}	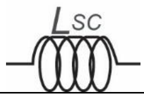	$L_{SC}\ (Ns^{2}m^{-5})$	0.70×10^{7}	$\mathbf{1.00 \times 10^{7}}$	3.20×10^{7}
*Reverse middle ear Z_{ME}	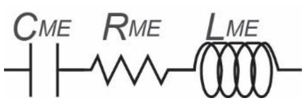	$C_{ME}\ (N^{-1}m^{5})$	11.30×10^{-15}	$** \mathbf{2.56 \times 10^{-15}}$	1.01×10^{-15}
		$R_{ME}\ (Nsm^{-5})$	2.74×10^{10}	$** \mathbf{4.76 \times 10^{10}}$	8.40×10^{10}
		$L_{ME}\ (Ns^{2}m^{-5})$	8.68×10^{5}	$** \mathbf{7.78 \times 10^{5}}$	8.60×10^{5}
*Vestibular aqueduct Z_{VA}	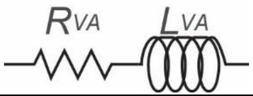	$R_{VA}\ (Nsm^{-5})$	0.43×10^{11}	$** \mathbf{0.98 \times 10^{11}}$	1.18×10^{11}
		$L_{VA}\ (Ns^{2}m^{-5})$	4.70×10^{6}	$** \mathbf{5.00 \times 10^{6}}$	2.00×10^{6}

* Parameters are fitted to experimental data measured from previous studies – not the same set of temporal bones of the present study.
** Parameters are fitted to the average measurements instead of median.

Simulate effects of SCD on AC-evoked intracochlear pressures

We used the AC inner-ear model of Fig. S2 and the median impedance parameters Z_{RW}, Z_{DIFF}, and Z_{SC} described in Table S1 to simulate P_{SV}/U_{STAP}, P_{ST}/U_{STAP}, and P_{DIFF}/U_{STAP} with and without SCD in Fig. S4. The simulations for the normal inner ear are plotted as solid black lines; the simulations of an ear with SCD are plotted with dashed black lines. These predictions are similar to our temporal bone experimental results plotted in Fig. S1.

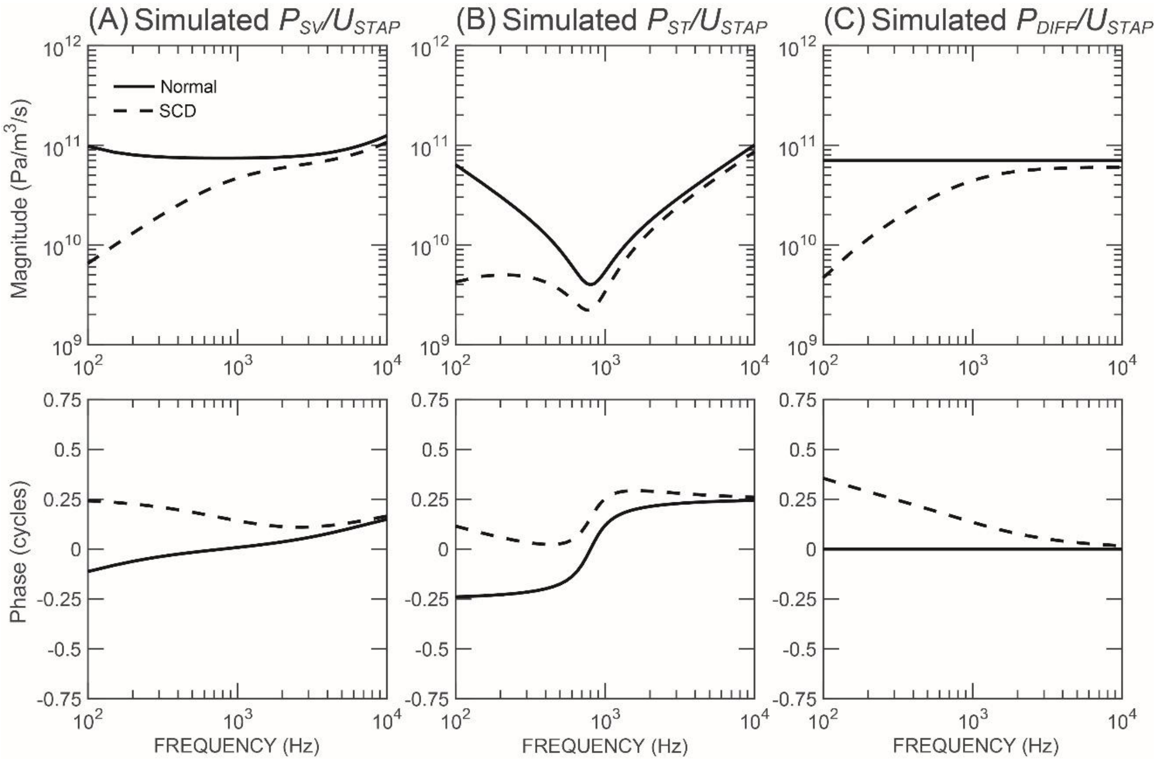

Figure S4. Simulated magnitude and phase of (A) P_{SV}/U_{STAP}, (B) P_{ST}/U_{STAP}, and (C) P_{DIFF}/U_{STAP} during AC under normal (solid lines) and SCD (dashed lines) conditions.

It should be noted that the AC model in Fig. S2 does not include any natural inner-ear leakage channels such as the vestibular aqueduct and cochlear aqueduct. We assume that the acoustic impedances of those inner-ear leakage channels are too high to greatly influence the sound transmission in the inner ear during AC over the tested frequency range. This assumption is based on evidence of equal volume velocity for round and oval windows during AC [11].

Bone Conduction

Temporal bone intracochlear sound pressures during BC.

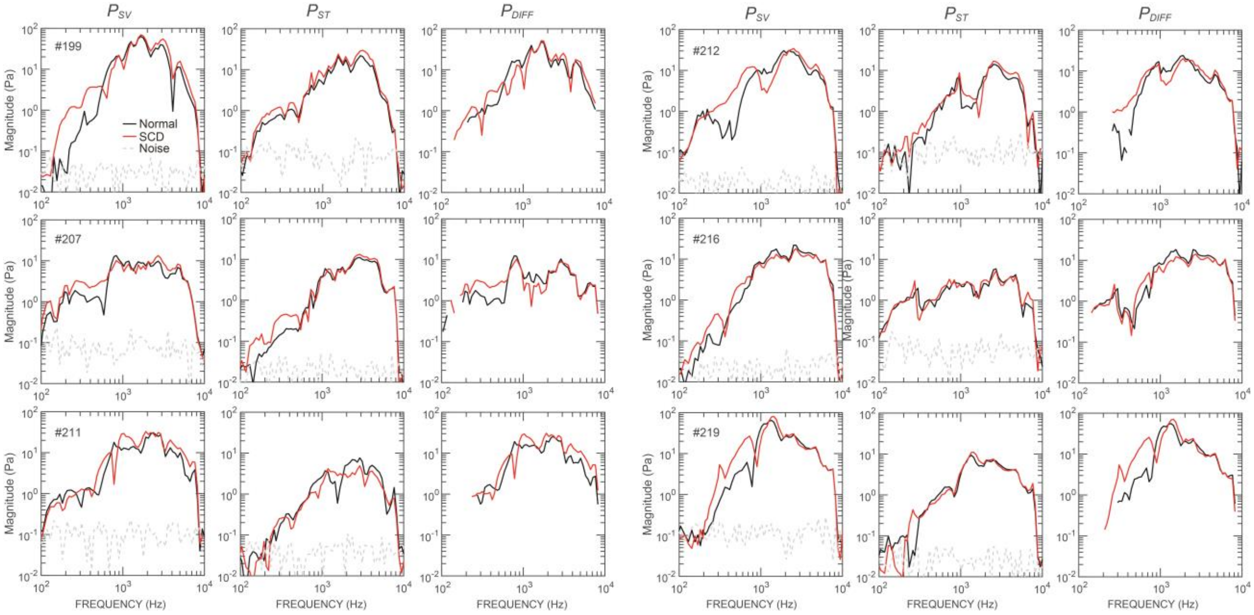

Figure S5. Magnitude frequency response of P_{SV}, P_{ST}, and P_{DIFF} measured in 6 ears in response to BC stimulation generated by a Baha BP100 driven by a sinusoidal voltage having a magnitude of 40 mV_{RMS}. Black lines plot the pressures under the normal condition; red lines plot the pressures after SCD; grey dashed lines plot the noise floors of the pressure sensors.

Outer and middle-ear BC mechanisms

The outer ear can contribute to BC hearing by expansion and compression of the compliant cartilaginous wall of the lateral part of the ear canal wall. The middle ear can contribute to BC hearing by the inertia of the ossicles [12]. The sound produced by these two BC mechanisms enters the inner ear via a pathway similar to that during AC. If SCD affects either of these BC mechanisms, SCD should then have similar effects for AC and BC sound transmission. However, SCD induces almost opposite effects for AC and BC: AC hearing decreases and BC hearing increases at low frequencies. Our intracochlear pressure measurements are consistent with these clinical findings. Therefore, the effect of SCD on BC sound transmission does not act through the outer- or middle-ear BC mechanisms.

Inner-ear bone compression model for BC.

Expansion and compression of the bony wall of the inner ear has been proposed as a mechanism for BC [7,13,14]. This concept is illustrated in Fig. S6A. In the normal ear, the bone vibration is believed to result in compression, squeezing the cochlear fluid towards the oval and the round windows (arrows in Fig. S6A), and then expanding. This would generate sound pressures near the oval window (P_{SV}) and round window (P_{ST}). Previously, based on this mechanism, we built a BC bone-compression model that resulted in predictions consistent with an increase in P_{DIFF} associated with SCD[7]. But that model was not tested against the individual effects on P_{SV} and P_{ST}. To quantitatively determine the SCD effects on P_{SV} and P_{ST} under the bone-compression mechanism, we re-created the BC bone-compression model as shown in Fig. S6B.

The two volume velocity sources, $\boldsymbol{U_{SV}}$ and $\boldsymbol{U_{ST}}$, represent the volume velocities generated by the change in SV space and the ST space, respectively, during volume compression and expansion. The impedances in this model are the same as those obtained from AC experimental data described above and in supplementary Table S1. Because U_{SV} and U_{ST} during bone compression are unknown, we try different ratios of U_{SV}/U_{ST} (from 0.1 to 10), to test if the simulated effects of SCD fit our experimental results.

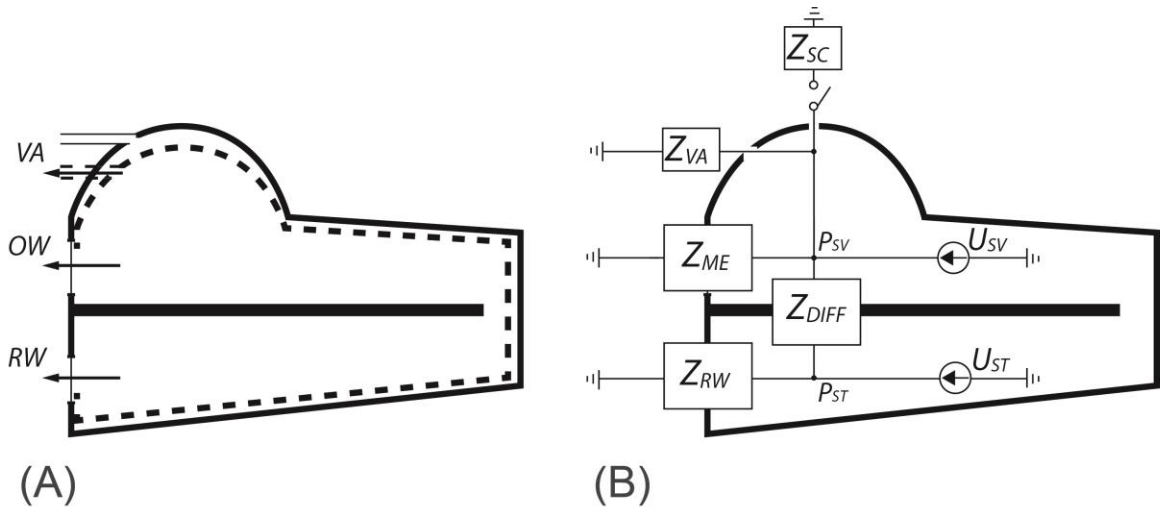

Figure S6. BC bone-compression model of the inner ear: (A) Concept of BC mechanism due to compression of the inner-ear wall. Dashed-line boundary represents the inner-ear wall during the compression. Arrows indicate volume velocity flows produced by the compression. (B) Lumped-

element model representation for sound transmission due to compression of the inner-ear wall during BC.

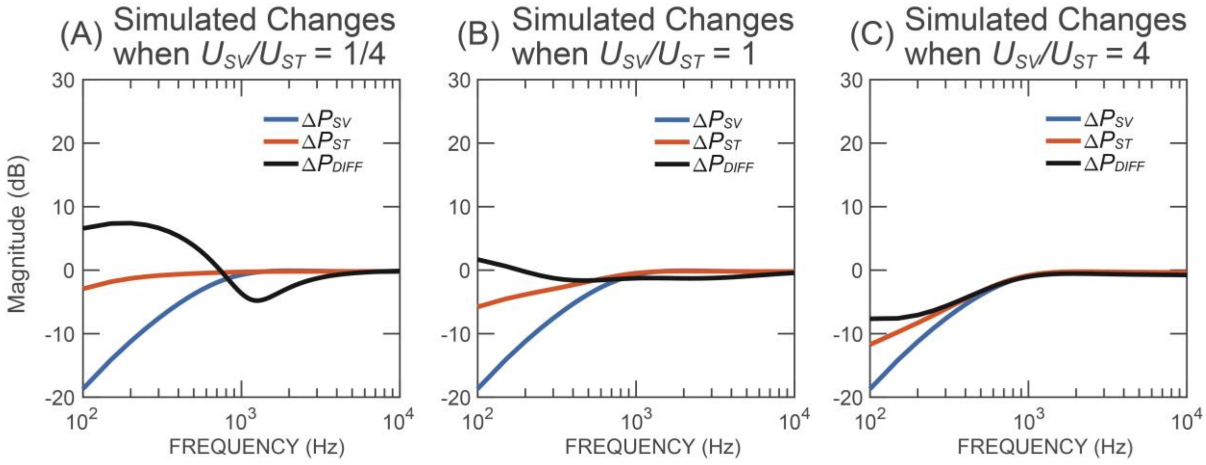

Figure S7. BC bone-compression model simulation results. SCD-induced changes in magnitude of P_{SV} (blue line), P_{ST} (orange line), and P_{DIFF} (black line) simulated by the bone-compression model when U_{SV}/U_{ST} is (A) 1/4, (B) 1/1, and (C) 4/1.

With the bone-compression model, SCD can increase P_{DIFF} at low frequencies (if $U_{SV}/U_{ST} \leq$ ¼, as shown in Fig. S7A). However, SCD decreases both P_{SV} and P_{ST} at low frequencies. This is inconsistent with temporal bone experimental results where SCD increases P_{SV} at low frequencies and has limited effect on P_{ST}, as shown in Fig. 2 in the main article. Thus, we conclude that the inner-ear wall compression mechanism is inconsistent with the SCD effects on BC sound transmission.

Effects of parameter variation on the BC inertia model

The Supplemental Figs. S8-S12 below show the simulated changes in P_{SV}, P_{ST}, and P_{DIFF} due to SCD in the BC inertia model (Fig. 3C) as the impedances in the model are varied, one at a time, from the minimum to the maximum (see Supplemental Table S1) while the other impedances were held at their median or average values.

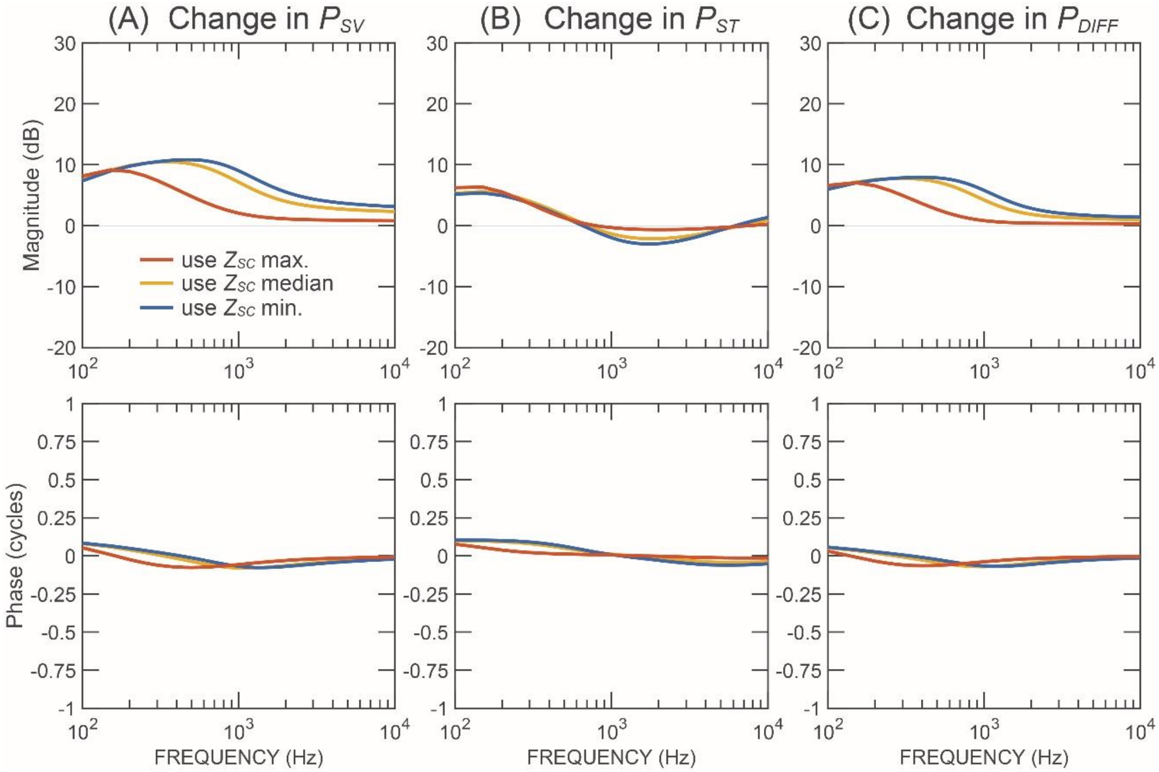

Figure S8. Simulated changes in P_{SV}, P_{ST}, and P_{DIFF} due to SCD in the BC inertia model when Z_{SC} is set at the minimum, median, and maximum values listed in Supplemental Table S1.

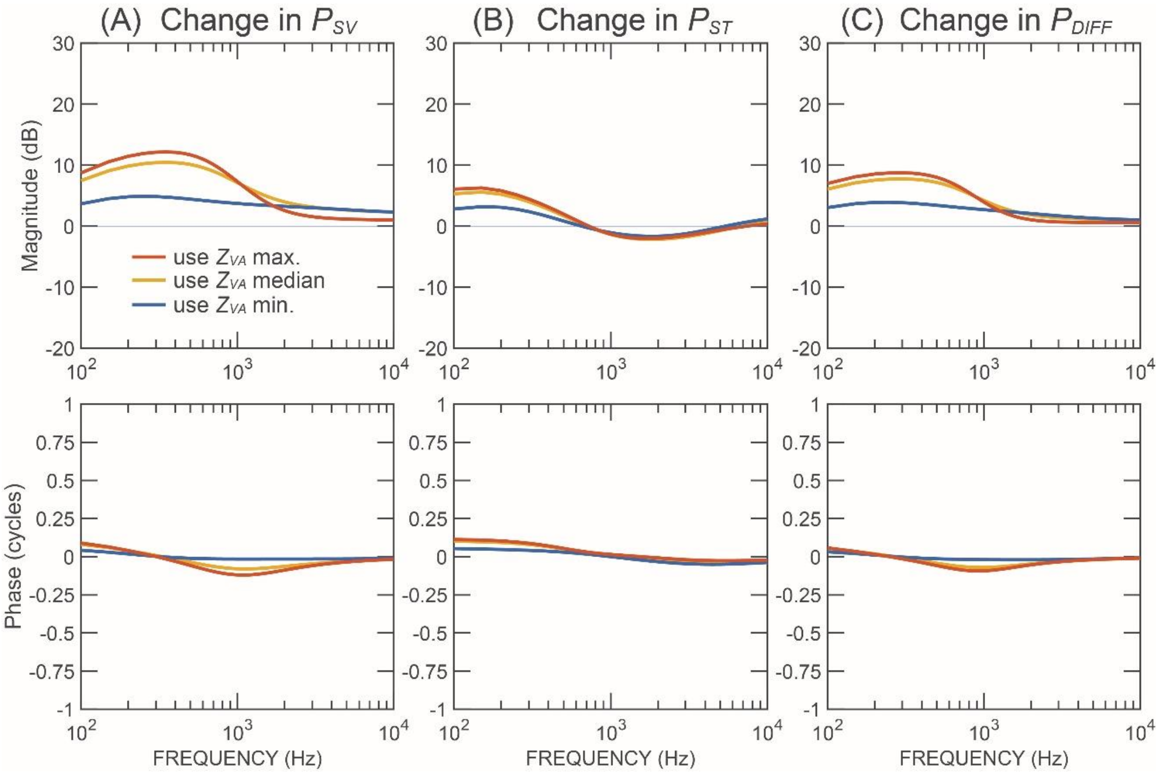

Figure S9. Simulated changes in P_{SV}, P_{ST}, and P_{DIFF} due to SCD in the BC inertia model when Z_{VA} is set at the minimum, median, and maximum values listed in Supplemental Table S1.

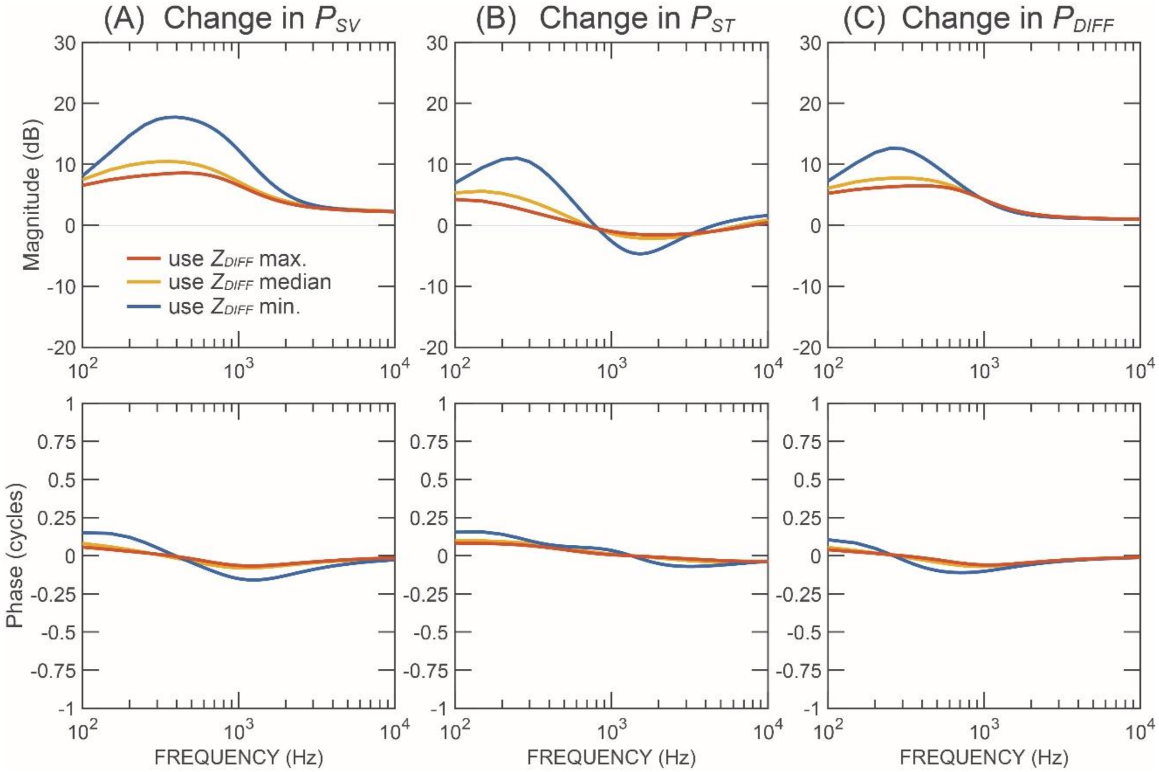

Figure S10. Simulated changes in P_{SV}, P_{ST}, and P_{DIFF} due to SCD in the BC inertia model when Z_{DIFF} is set at the minimum, median, and maximum values listed in Supplemental Table S1.

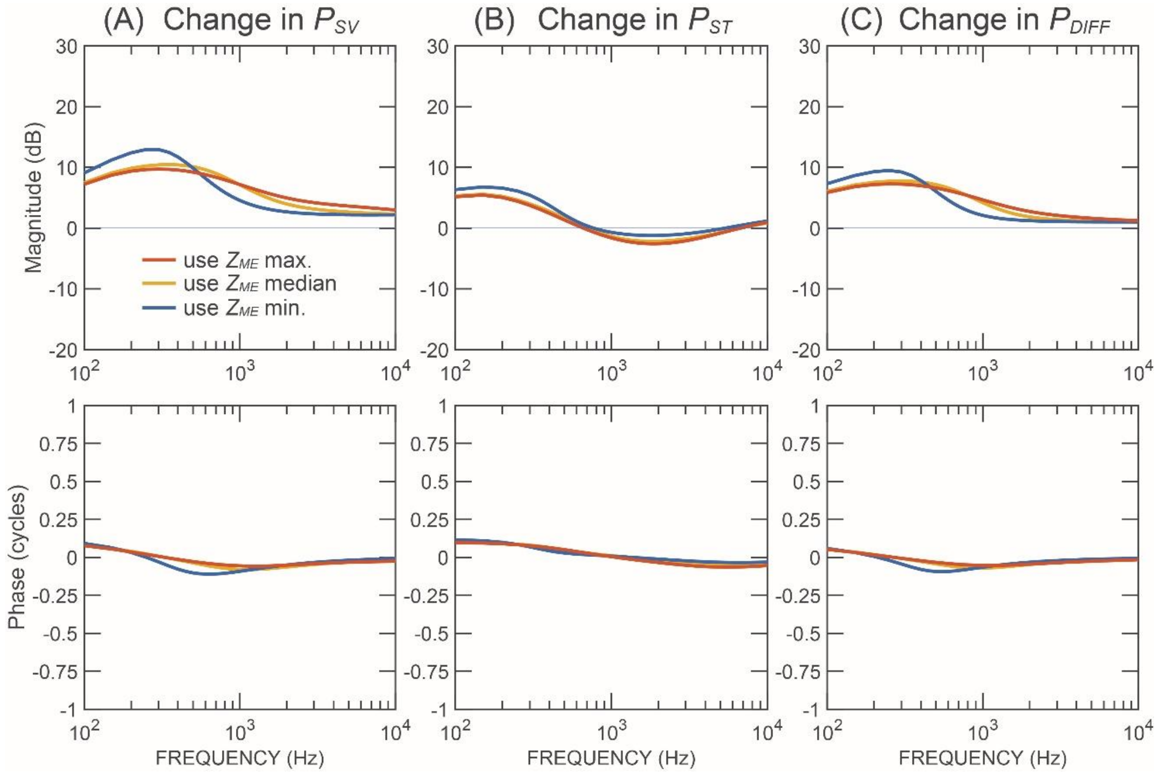

Figure S11. Simulated changes in P_{SV}, P_{ST}, and P_{DIFF} due to SCD in the BC inertia model when Z_{ME} is set at the minimum, median, and maximum values listed in Supplemental Table S1.

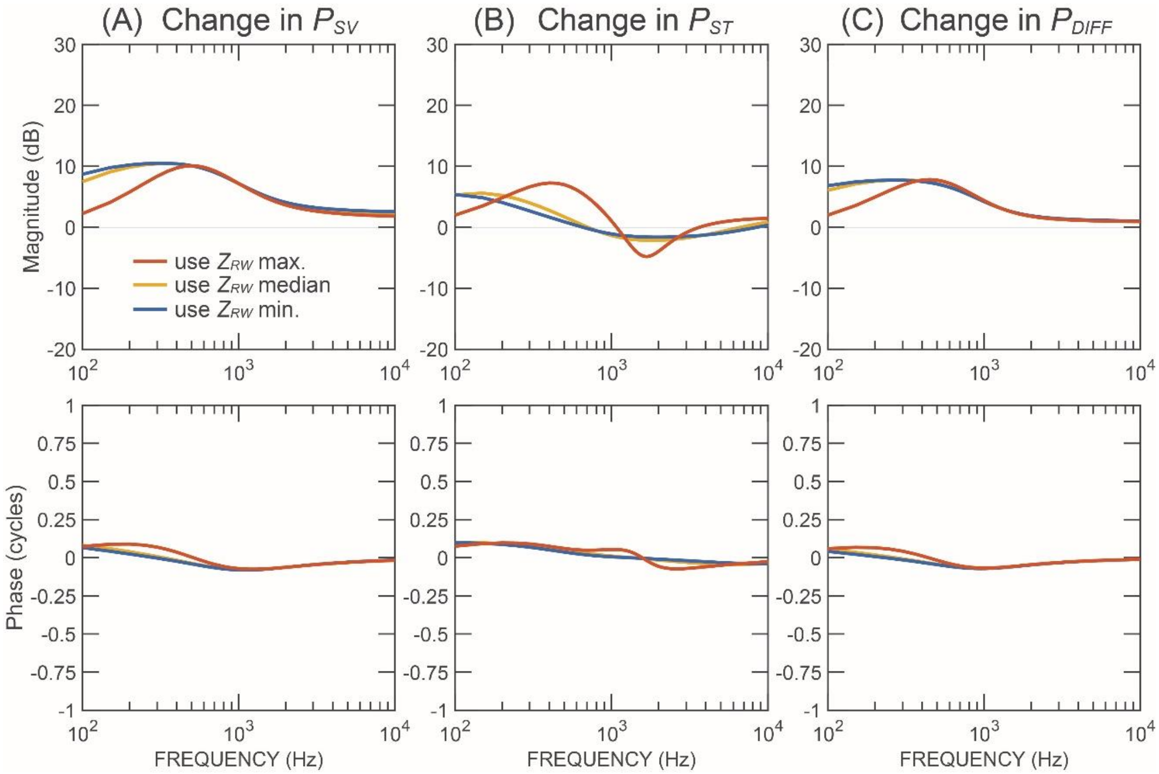

Figure S12. Simulated changes in P_{SV}, P_{ST}, and P_{DIFF} due to SCD in the BC inertia model when Z_{RW} is set at the minimum, median, and maximum values listed in Supplemental Table S1.

Prediction of the effect of SCD size and location on BC hearing

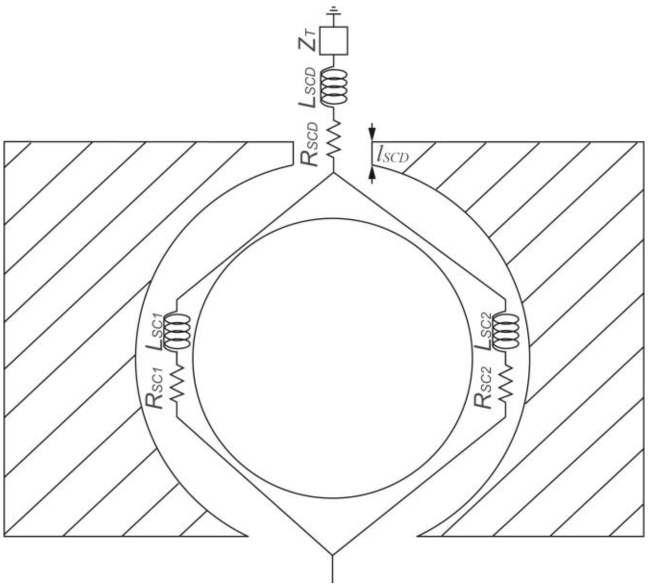

Figure S13. Structure based acoustic circuit representing a superior semicircular canal with an SCD. Z_T represents the termination impedance interfacing the SCD.

Figure S13 shows an acoustic circuit of the superior canal with an SCD. The two arms of the superior canal and the SCD are treated as tubes of small diameters. The resistors (R_{SC}) and inductors (L_{SC}) represent the acoustic resistance and mass associated with the water-like lymph in the tube, with values that depend on the length and diameter of the tube (Eq. 5.48 and 5.49 in Beranek). Z_T is the termination impedance at the SCD interface. If the SCD interfaces the brain, Z_T represents the impedance of the fluid- and tissue-filled cranial vault; if the SCD interfaces the air-filled middle ear cavity, Z_T represents the impedance of the air in the cavity.

Table S2 summarizes the parameters used to calculate the theoretical impedance of the superior canal (Z_{SC_Theo}), the theoretical impedance of a circular SCD of various diameters from 0.1 mm to 1 mm (Z_{SCD_Theo}), and the termination impedance (Z_T) for the two conditions - the middle ear cavity and the cranial vault.

The total impedance of the superior canal branch, Z_{SC_Total}, is determined by

$$Z_{SC\_Total} = Z_{SC\_Theo} + Z_{SCD\_Theo} + Z_T$$

Table S2. Dimensions and equations used to calculate the impedances in Figure S13.

Impedance	Dimensions	Element type and relation to dimensions
Superior canal Z_{SC_Theo}	Length of each arm $l_{SC} = 5\ mm$<br>Radius of the inner wall of the canal $a_{SC} = 0.4\ mm$	$* R_{SC1} \& R_{SC2} = \frac{8\eta l_{SC}}{\pi a_{SC}^4}$
		$* L_{SC1} \& L_{SC2} = \frac{4\rho l_{SC}}{3\pi a_{SC}^2}$

Superior canal dehiscence Z_{SCD_Theo}	Thickness of the wall of the superior canal $l_{SCD} = 0.25\ mm$<br>Radius of the dehiscence a_{SCD} varying from $0.05\ mm$ to $0.5\ mm$	$R_{SCD} = \frac{8\eta l_{SCD}}{\pi a_{SCD}^4}$<br>$L_{SCD} = \frac{4\rho l_{SCD}}{3\pi a_{SCD}^2}$
Termination Z_T – Cranial space	Volume of cranial vault $V = 1\ liter$ (filled with water)	$*\ C_{CS} = \frac{\rho}{Vc^2}$
Termination Z_T – Middle ear air space	Estimated by a four-component network and detailed in Rosowski and Merchant 1994	

Other constants used in the calculation: η – dynamic viscosity of water $1.002 \times 10^{-3} kg/(m \cdot s)$; ρ – density of water $1000\ kg/m^3$; c – speed of sound in water 1480 m/s.
* From equation 5.48 & 5.49 in Beranek.

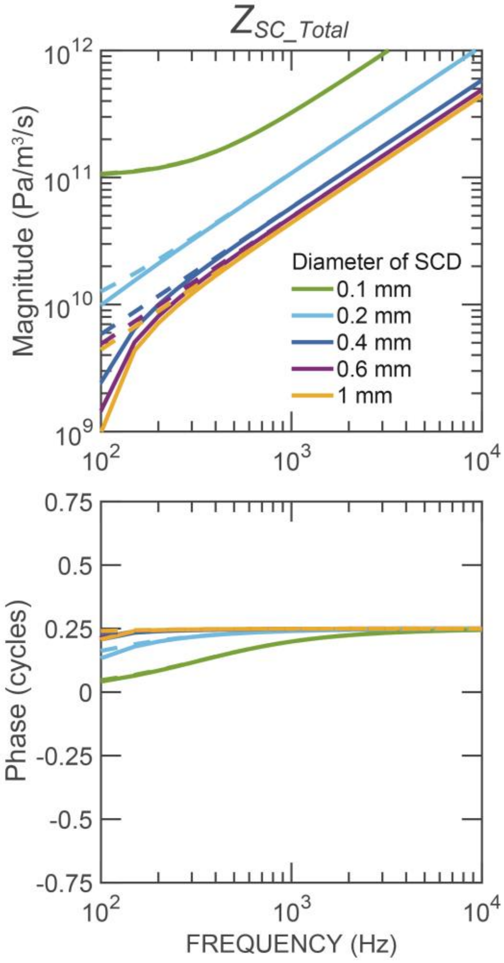

Figure 14. Theoretically derived magnitude and phase of Z_{SC_Total} with different sizes of SCD and different types of termination impedance. The solid color lines represent the Z_{SC_Total} of various diameters of SCD facing the cranial vault. The dashed color lines represent the Z_{SC_Total} of various diameters of SCD facing the middle ear cavity air.

Figure S14 plots Z_{SC_Total} with different sizes of SCD and different terminating conditions. When the dehiscence is extremely small (diameter of 0.1 mm), Z_{SC_Total} is relatively high and is dominated by Z_{SCD_Theo}. As the size of the dehiscence increases, Z_{SCD_Theo} decreases and so does Z_{SC_Total}. When the diameter of SCD ≥ 0.4 mm, Z_{SC_Total} is dominated by Z_{SC_Theo}, and changes little with further increases in the size of the SCD.

When the diameter of the dehiscence ≤ 0.2 mm, the Z_{SC_Total} is insensitive to Z_T because Z_{SC_Total} is dominated by Z_{SCD_Theo}. For SCDs of diameters ≥ 0.4 mm when the dehiscence is terminated by the cranial vault, Z_{SC_Total} are smaller at f < 150 Hz, due to a resonance between Z_{SC_Theo} and Z_T.

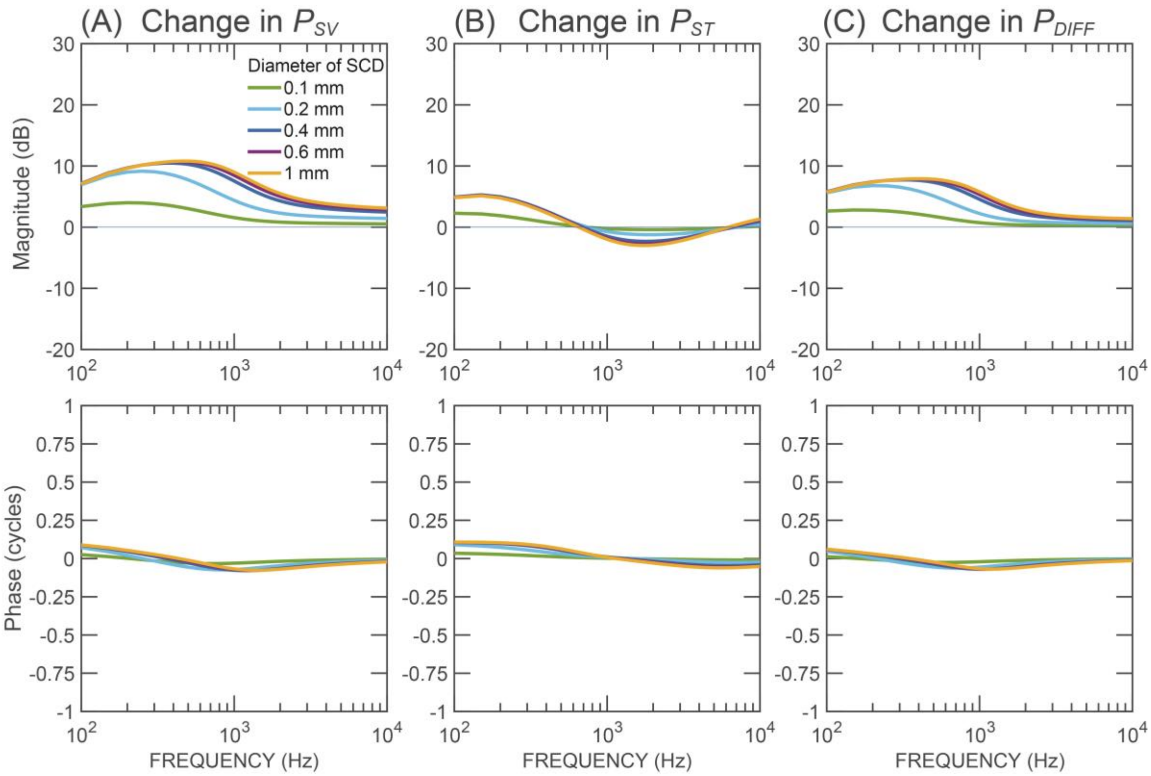

Figure S15. Simulated effects of SCD size on the changes of P_{SV}, P_{ST}, and P_{DIFF} in the BC inertia model when the SCD faces the cranial vault.

To study the effects of SCD size and the different termination conditions on BC-elicited intracochlear pressures, we replaced Z_{SC} with Z_{SC_Total} in the BC inertia model in Fig. 3C. Figure S15 shows the predicted changes of P_{SV}, P_{ST}, and P_{DIFF} with varying diameter of the SCD when the dehiscence faces the cranial vault. As the size of SCD increases, the low-frequency increases in P_{SV}, P_{ST}, and P_{DIFF} become more prominent. However, when the SCD diameter is 0.4 mm or greater, the changes in P_{SV}, P_{ST}, and P_{DIFF} are insensitive to the size of the dehiscence.

When we change the termination impedance from the cranial space to the middle ear cavity, the changes in P_{SV}, P_{ST}, and P_{DIFF} for a given size of the SCD remain the same (therefore simulation results are not shown). The model suggests that BC hearing would increase by the same amount due to the SCD regardless of which space the SCD interfaces.

Experimental protocol

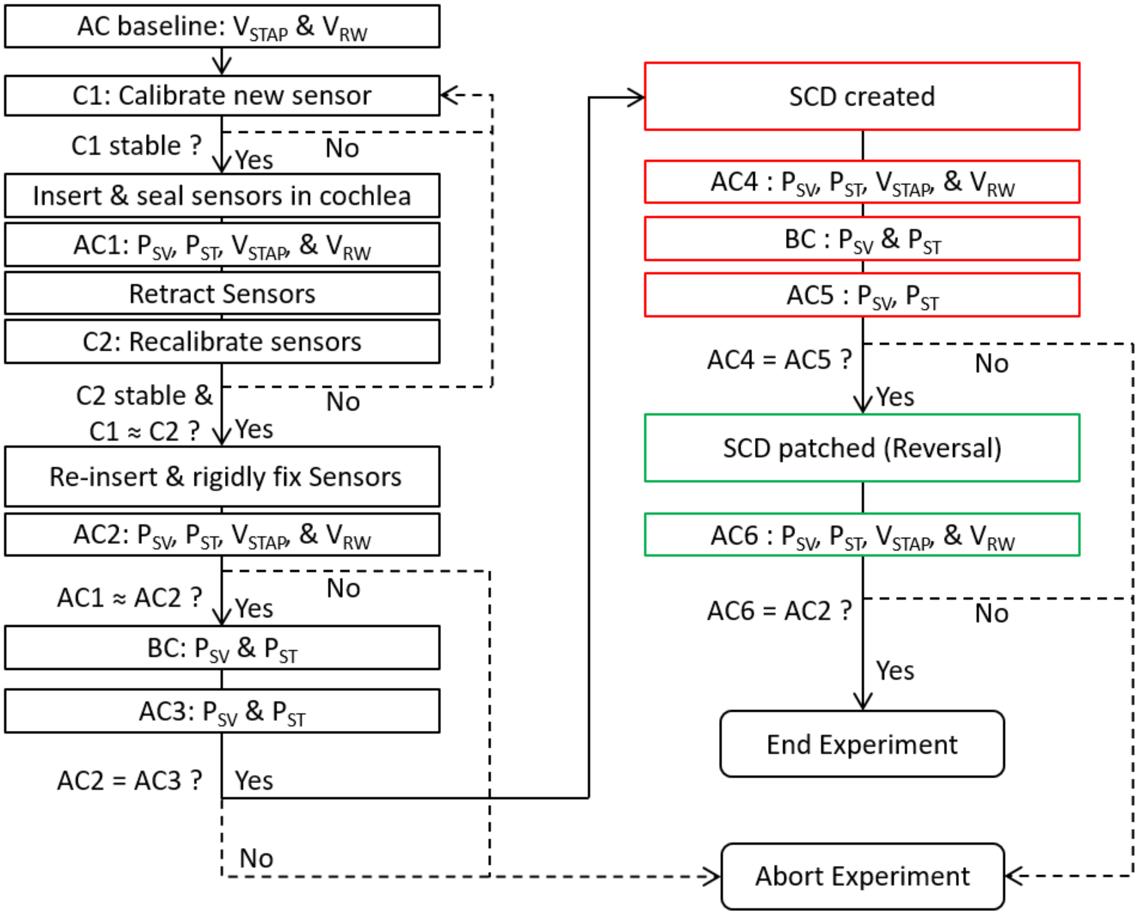

Figure S16. Flow chart of the experimental protocol.

References for supplementary information

1 Cheng, Y. S. *et al.* Superior Canal Dehiscence Similarly Affects Cochlear Pressures in Temporal Bones and Audiograms in Patients. *Ear Hear*, doi:10.1097/AUD.0000000000000799 (2019).

2 Niesten, M. E. *et al.* Assessment of the effects of superior canal dehiscence location and size on intracochlear sound pressures. *Audiol Neurootol* **20**, 62-71, doi:10.1159/000366512 (2015).

3 Pisano, D. V., Niesten, M. E., Merchant, S. N. & Nakajima, H. H. The effect of superior semicircular canal dehiscence on intracochlear sound pressures. *Audiol Neurootol* **17**, 338-348, doi:10.1159/000339653 (2012).

4 Frear, D. L., Guan, X., Stieger, C., Rosowski, J. J. & Nakajima, H. H. Impedances of the inner and middle ear estimated from intracochlear sound pressures in normal human temporal bones. *Hear Res* **367**, 17-31, doi:10.1016/j.heares.2018.06.019 (2018).

5 Nakajima, H. H. *et al.* Differential intracochlear sound pressure measurements in normal human temporal bones. *Journal of the Association for Research in Otolaryngology : JARO* **10**, 23-36, doi:10.1007/s10162-008-0150-y (2009).

6 Raufer, S., Masud, S. F. & Nakajima, H. H. Infrasound transmission in the human ear: Implications for acoustic and vestibular responses of the normal and dehiscent inner ear. *J Acoust Soc Am* **144**, 332, doi:10.1121/1.5046523 (2018).

7 Rosowski, J. J., Songer, J. E., Nakajima, H. H., Brinsko, K. M. & Merchant, S. N. Clinical, experimental, and theoretical investigations of the effect of superior semicircular canal dehiscence on hearing mechanisms. *Otology & neurotology : official publication of the American Otological Society, American Neurotology Society [and] European Academy of Otology and Neurotology* **25**, 323-332 (2004).

8 Elliott, S. J., Ni, G. & Verschuur, C. A. Modelling the effect of round window stiffness on residual hearing after cochlear implantation. *Hear Res* **341**, 155-167, doi:10.1016/j.heares.2016.08.006 (2016).

9 Xue, L. *et al.* The role of third windows on human sound transmission of forward and reverse stimulations: A lumped-parameter approach. *J Acoust Soc Am* **147**, 1478, doi:10.1121/10.0000846 (2020).

10 Stieger, C., Rosowski, J. J. & Nakajima, H. H. Comparison of forward (ear-canal) and reverse (round-window) sound stimulation of the cochlea. *Hear Res* **301**, 105-114, doi:10.1016/j.heares.2012.11.005 (2013).

11 Stenfelt, S., Hato, N. & Goode, R. L. Fluid volume displacement at the oval and round windows with air and bone conduction stimulation. *J Acoust Soc Am* **115**, 797-812 (2004).

12 Stenfelt, S. & Goode, R. L. Bone-conducted sound: physiological and clinical aspects. *Otol Neurotol* **26**, 1245-1261 (2005).

13 Kirikae, I. An experimental study on the fundamental mechanism of bone conduction. *Acta Otolaryngol Suppl* **145**, 1-111 (1959).

14 Stenfelt, S. Inner ear contribution to bone conduction hearing in the human. *Hear Res* **329**, 41-51, doi:10.1016/j.heares.2014.12.003 (2015).
